# The cardiac diagnostic work-up in stroke patients—A subanalysis of the Find-AF_RANDOMISED_ trial

**DOI:** 10.1371/journal.pone.0216530

**Published:** 2019-05-09

**Authors:** Katrin Wasser, Mark Weber-Krüger, Falko Jürries, Jan Liman, Gerhard F. Hamann, Pawel Kermer, Timo Uphaus, Evgeny Protsenko, Joachim Seegers, Meinhard Mende, Klaus Gröschel, Rolf Wachter

**Affiliations:** 1 Clinic for Neurology, University of Göttingen, Göttingen, Germany; 2 Clinic for Cardiology and Pneumology, University of Göttingen, Göttingen, Germany; 3 Clinic for Neurology and Neurorehabilitation, Bezirkskrankenhaus Günzburg, Günzburg, Germany; 4 Clinic for Neurology, Nordwest-Krankenhaus Sanderbusch, Sande, Germany; 5 Clinic and Polyclinic for Neurology, University of Mainz, Mainz, Germany; 6 Department of Internal Medicine II, Division of Cardiology, University Hospital Regensburg, Regensburg, Germany; 7 Institute for Medical Informatics, Statistics and Epidemiology (IMISE), University Hospital Leipzig, Leipzig, Germany; 8 Clinic and Policlinic for Cardiology, University Hospital Leipzig, Leipzig, Germany; 9 DZHK (German Centre for Cardiovascular Research), partner site Göttingen, Germany; Public Library of Science, UNITED KINGDOM

## Abstract

**Background:**

The cardiac diagnostic workup of stroke patients, especially the value of echocardiography and enhanced and prolonged Holter-ECG monitoring, is still a matter of debate. We aimed to analyse the impact of pathologies detected by echocardiography and ECG monitoring on therapeutic decisions and prognosis.

**Methods:**

Find-AF_RANDOMISED_ was a prospective multicenter study which randomised 398 acute ischemic stroke patients ≥ 60 years to enhanced and prolonged Holter-ECG monitoring or usual stroke unit care. This substudy compared therapeutic consequences of echocardiography and routine Holter-ECG or enhanced and prolonged Holter-ECG monitoring, respectively, and prognosis of patients with or without pathologic findings in echocardiography or Holter-ECG monitoring.

**Results:**

50.3% received enhanced and prolonged Holter-ECG monitoring and 49.7% routine ECG monitoring. 82.9% underwent transthoracic echocardiography (TTE), 38.9% transesophageal echocardiography (TEE) and 25.6% both procedures. 14/89 TEE pathologies and 1/90 TTE pathology led to a change in therapy, resulting in a number needed to change decision (NNCD) of 12 and 330 (p < 0.001), respectively. In comparison, enhanced and prolonged Holter-ECG monitoring found atrial fibrillation (AF) in 27 of 200 patients, and routine ECG monitoring in twelve of 198 patients, leading to therapeutic changes in all patients (NNCD 8 and 17, respectively, p < 0.001).

**Conclusions:**

Most changes in therapeutic decisions were triggered by enhanced and prolonged Holter-ECG monitoring, which should therefore play a more prominent role in future guidelines. Echocardiography identifies a patient group at high cardiovascular risk, but rarely result in therapeutic changes. Whether this patient group requires further cardiovascular workup remains unknown. This should be further investigated by interdisciplinary neurocardiologic teams and in appropriate future trials.

**Trial registration:**

ClinicalTrials.gov NCT01855035

## Introduction

Ischemic stroke is one of the most common causes of disability and death worldwide and the 5-year-recurrence rate after a first brain ischemia is up to 30% [1]. Therefore, a careful diagnostic workup is needed to exclude potential stroke mechanisms that may result in therapeutic changes. The American Heart Association/American Stroke Association (AHA/ASA) and the European Stroke Organisation (ESO) agree on a standard of care, which includes continuous monitoring of vital parameters and neurologic status, systematic laboratory testing, cerebral imaging, extra- and transcranial Doppler and duplex sonography, 12-lead ECG and a minimum of 24-hour ECG-monitoring [2,3].

These recommendations of a relatively short period of ECG monitoring of these two most cited guidelines are in contrast to the well-documented fact that enhanced and prolonged Holter-ECG monitoring significantly improves the detection rate of paroxysmal AF [4–6], which is of crucial importance as it usually shifts the secondary prevention therapy from antiplatelet drugs to oral anticoagulation (OAC) that is known to reduce the stroke risk by up to 64% on an intention-to treat basis [7]. In contrast, the European Society of Cardiology (ESC) and a consensus document of German cardiologists and neurologists recommend a duration of > 72 hours for Holter-ECG monitoring after acute ischemic stroke [8,9].

It is uncontroversial that echocardiography can detect many potential cardiac sources of embolism such as left atrial thrombus, patent foramen ovale, atrial septum aneurysm, valvular or myocardial disease, endocarditis or cardiac tumors amongst others. Furthermore, it can reveal other cardiac pathologies of potential therapeutic consequences such as wall motion abnormalities or a reduced left ventricular function which potentially demands a change in cardiologic therapy. Despite the fact that echocardiography can provide useful information, the indication and optimal echocardiographic approach in the cardiac workup of ischemic stroke are still unclear and not specifically addressed in current AHA/ASA guidelines [3,10]. The criteria for stroke unit certification by European Stroke Organisation (ESO) and German Stroke Society (DSG) have required the availability of TTE and TEE [11]. TTE is generally available, non-invasive, less personnel-intensive and cheap, whereas TEE is superior for evaluation of the aortic arch, left atrium, and atrial septum [12–14]. Therefore, DSG demands a minimum rate of 15% TEE of all stroke unit patients [15]. Potential therapeutic consequences of pathologic echocardiographic findings vary from conservative therapies such as OAC or antibiotics to percutaneous coronary or surgical intervention according to current guidelines. But the net clinical benefit of TEE in stroke patients (i.e. how often does TEE lead to a change in therapeutic management) is still unknown. Current literature investigated mostly only the impact of echocardiographic pathologies in subgroups of stroke like cryptogenic stroke or ESUS [16,17]. Accordingly, there are no specific recommendations for the use of echocardiography in other stroke subgroups in current guidelines [3,10]. Most therapeutic strategies mentioned in guidelines are only limited to the treatment of cardiac sources of embolism and there is a lack of clear strategies for the treatment of pathologies that are not directly linked to stroke, Therefore, therapeutic decisions based on echocardiographic findings in stroke patients often seem to underlie local in-house policies or are reduced to the treatment of pathologies that are directly linked to stroke like cardiac sources of embolism, instead of treating all pathologies that affect the well-being and survival or simply the prognosis of stroke patients.

In this subanalysis of the Find-AF_RANDOMISED_ trial we aimed to analyse the impact of pathologies detected by echocardiography and ECG monitoring on therapeutic decisions and prognosis. We therefore investigated the number and types of pathologies detected by TTE or TEE, routine or enhanced and prolonged Holter-ECG monitoring and analysed how often these findings led to changes in neurologic or cardiologic therapeutic decisions. In addition, we evaluated whether abnormal findings in both modalities (echocardiography and Holter-ECG monitoring) may predict a worse prognosis represented by an increased one-year mortality in comparison to patients without pathologies in echocardiography or routine Holter-ECG or enhanced and prolonged Holter-ECG monitoring, respectively.

## Methods

### Study design and patient population

The local ethics committees (Göttingen, Mainz, Wiesbaden and Sanderbusch, Germany) approved the protocol of the Find-AF_RANDOMISED_ trial and all patients gave written informed consent. Find-AF_RANDOMISED_ was an investigator-initiated prospective, randomised, controlled, open-label multicenter study that has been described in detail, recently (S1 Fig) [4,16]. In brief, we included patients ≥ 60 years with acute ischemic stroke irrespective of the stroke aetiology. Exclusion criteria were a history or presence of AF, an ipsilateral carotid artery stenosis ≥ 50% according to NASCET and an indication or contraindication for OAC.

Within seven days after an acute ischemic stroke, patients were admitted to a certified stroke unit and received the routine aetiologic stroke workup including transthoracic and/or transesophageal echocardiography according to local in-house policies. Patients were either randomised to 30 total days of Holter-ECG-monitoring (10 days at randomization, after three and after six months) or standard-of-care monitoring with 24-hour Holter- and/or telemetry ECG according to local stroke unit protocols. All AF episodes were adjudicated by a blinded endpoint committee and AF was defined as at least one episode ≥ 30 seconds duration [6]. Furthermore, all ECGs were screened for potential pacemaker indications, e.g. high-grade atrioventricular blocks or extreme bradycardia amongst others. After randomisation to the two ECG groups almost all study patients received either transthoracic or transesophageal echocardiography or both. The type of echocardiography was chosen according to local in-house policies. Echocardiography was performed by investigators with ≥ 2 years of experience according to the current guideline for the use of echocardiography in the evaluation of a cardiac source of embolism [17]. The echocardiographers did not participate in any study specific training and used classifications according to local standards. Pathologic echocardiographic findings, which typically lead to a therapeutic consequence for patients, were a priori determined by an expert panel of cardiologists (R.W. and M. W.-K.). High-risk sources according to the TOAST classification were left atrial (appendage) and ventricular thrombus, myxoma and endocarditis. Medium-risk sources were patent foramen ovale (PFO) and atrial septal aneurysm (ASA) [18]. Other echocardiographic findings considered to be relevant were other severe valve diseases, ventricular or aortic aneurysm, aortic plaques, impaired left ventricular ejection fraction (LVEF) and wall motion abnormalities (Table 1). All pathologies were prospectively recorded into a predefined case report form (CRF). Potential therapeutic consequences such as OAC, interventions or operations were assessed during the clinical follow-ups after three, six and twelve months. Furthermore, adverse events, hospital admissions, diseases, and medication were recorded.

**Table 1:** Echocardiographic pathologies and potential therapeutic consequences.

pathology	number of patients (TTE)	number of patients (TEE)	potential therapeutic consequences	number of patients treated	therapy decision based on TTE	therapy decision based on TEE
LA thrombus^1^	N/A	0	OAC	0	0	0
LAA thrombus^1^	N/A	1	OAC	1	0	1
LV thrombus^1^	0	N/A	OAC	0	0	0
LV aneurysm^2^	1	N/A	OAC	0	0	0
Myxoma^1^	N/A	1	Operation	0	0	0
Endocarditis^1^	N/A	0	Operation	0	0	0
			Antibiotics	0	0	0
PFO and/or ASA^2^	N/A	40	OAC	9	0	9
			PFO closure	1	0	1
Aortic aneurysm^2^	1	3	Operation	0	0	0
Aortic plaques^2^	0	34	OAC	1	0	1
Severe valve insufficiency*	1	0	Operation	0	0	0
			Intervention	0	0	0
Severe valve stenosis°^2^	2	0	Operation	1	1	0
			Intervention	0	0	0
Valve thrombosis^1^	0	2	OAC	1	0	1
			Operation	1	0	1
LVEF < 50%	27	N/A	PCI	0	0	0
			Bypass	0	0	0
Hypo- and/or akinesia	44	N/A	PCI	0	0	0
			Bypass	0	0	0

^1^ = high-risk sources of cardioembolism

^2^ = medium-risk sources of cardioembolism

LA = left atrium, LAA = left atrial appendage, LV = left ventricle

* Tricuspid valve insufficiency

° aortic valve stenosis

N/A = not applicable, PCI = percutaneous coronary intervention

### Statistics

Continuous values were expressed as mean +/- SD and nominal variables as count and percentages. Comparisons of means were realized by t-test for independent samples. Median values with the corresponding interquartile range (IQR) were computed for non-normally distributed variables. For comparisons of categorical data we used two-tailed Chi-square statistics with Yates’ correction and Fisher’s exact test as appropriate.

One-year survival data for patients with or without pathologic echocardiographic or ECG findings were depicted using the Kaplan-Meier method. To adjust for age and centre heterogeneity, we fitted mixed linear Cox model for time-to-event data with random intercept for centre.

For a straightforward interpretation we defined the measure "Number Needed to Change Decision" (NNCD) as the average number of patients to be diagnosed in order to encounter one for whom the treatment decision changes. NNCD is the inverse of the absolutefrequency. Nonparametric 95% confidence intervals were calculated and tests were performed by simulation (n = 10,000).

All statistical analyses were performed using SPSS version 23.0 and higher (SPSS, Inc.) and R. Significance level for two-tailed tests is defined 0.05.

## Results

Between May 8, 2013, and Aug 31, 2014, 2848 patients ≥ 60 years were admitted with an ICD diagnosis I 63.x (cerebral infarction) in the four study centres. Out of this screening population, 402 patients were enrolled and randomised to enhanced and prolonged Holter-ECG monitoring and to usual care (Holter-) ECG monitoring. Usual care included telemetry in 95% of patients and Holter-ECG in 75% of patients. Main exclusion criteria were history of AF or AF on admission ECG, ipsilateral ICA stenosis ≥ 50% according to NASCET criteria, and indication or contraindication for oral anticoagulation. Four patients were erroneously randomised because of unknown history of AF or a severe ipsilateral carotid artery stenosis and thus the data of 398 patients were finally analysed. 200 patients were randomised to prolonged Holter-ECG monitoring and 198 patients to standard-of-care monitoring.

### Percentage of patients receiving transthoracic and transesophageal echocardiography

**Fig 1:**
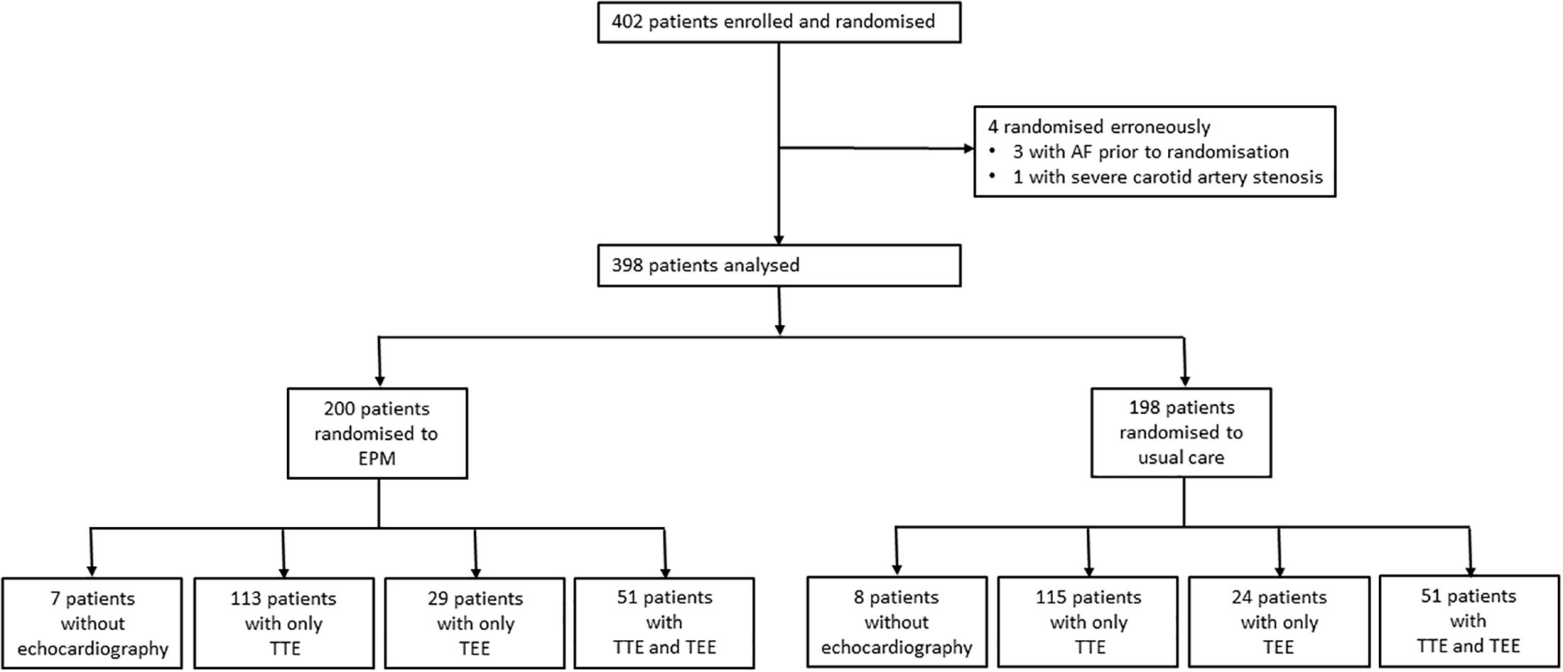
Trial profile of the present analysis.

TTE and TEE were performed according to local standards and the echocardiographic modalities were distributed approximately equally among the two ECG randomisation groups. 113 enhanced and prolonged Holter-ECG monitoring patients (56.5%) and 115 usual care patients (58.1%) received only TTE, 29 (14.5%) and 24 (12.1%) patients underwent only TEE and 51 patients of each group received both procedures (25.5% and 25.7%, respectively). Only 7 patients of the enhanced and prolonged Holter-ECG monitoring group (3.5%) and 8 patients of the usual care group (4.1%) received neither TTE nor TEE (Fig 1).

**Fig 2:**
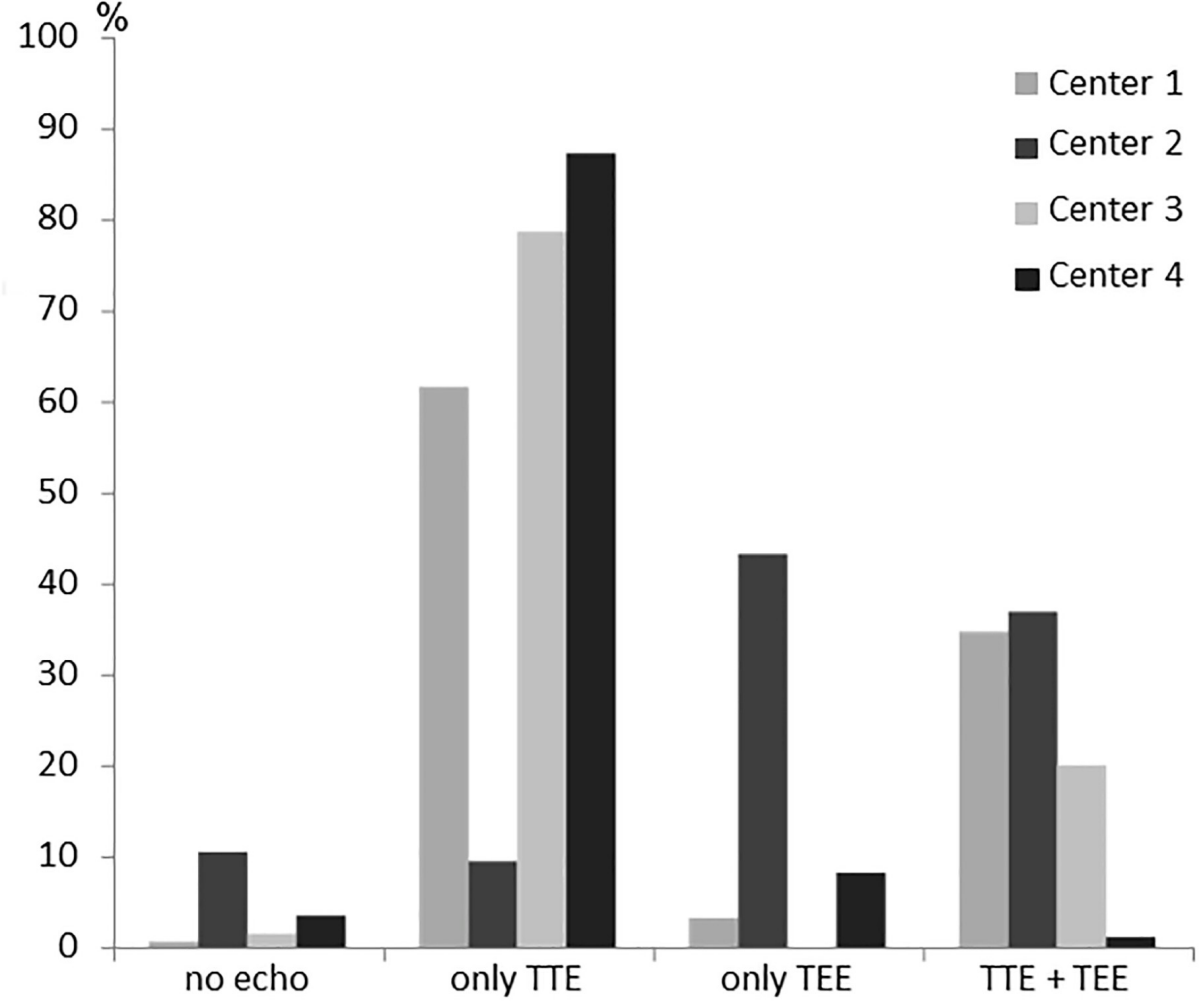
Rates of echocardiographies in each study centre.

The echocardiography rates differed between the four study centres, e.g. the percentage of patients receiving TEE ranged from 9 to 80% (Fig 2).

Baseline characteristics of all patients divided in the randomisation groups and subdivided in four patient groups without echocardiography, with only TTE or TEE or both procedures, respectively, are summarized in Table 2. The main difference was that a higher percentage of patients with cardioembolism received TEE as compared to patients with other stroke aetiologies (p < 0.001) and that TEE patients were significantly younger (p < 0.001).

**Table 2:** Baseline characteristics of the study population.

	Alln = 398	Enhanced and prolonged Holter-ECG monitoring, n = 200				Usual care, n = 198			
		no echocardio-graphy n = 7	only TTE done n = 113	only TEE done n = 29	TTE and TEE done n = 51	no echocardio-graphy n = 8	only TTE done n = 115	only TEE done n = 24	TTE and TEE done n = 51
Mean age (years)	72.7 (SD 7.5)	74.7 (SD 5.5)	74.5 (SD 7.1)	68.7 (SD 6.6)	68.5 (SD 6.6)	76.1 (SD 7.8)	74.8 (SD 7.4)	72.4 (SD 8.1)	69.7 (SD 6.4)
Female sex	160 (40.1%)	4 (57.1%)	51 (45.1%)	10 (34.5%)	20 (39.2%)	5 (62.5%)	45 (39.1%)	5 (20.8%)	20 (39.2%)
Medical history									
Art. hypertension	316 (79.2%)	6 (85.7%)	95 (84.1%)	21 (72.4%)	35 (68.6%)	8 (100.0%)	92 (80.0%)	17 (70.8%)	42 (82.4%)
Diabetes mellitus	108 (27.1%)	4 (57.1%)	37 (32.7%)	6 (20.7%)	9 (17.6%)	2 (25.0%)	28 (24.3%)	7 (29.2%)	15 (29.4%)
Hyperlipidemia	164 (41.1%)	3 (42.9%)	49 (43.4%)	5 (17.2%)	20 (39.2%)	4 (50.0%)	48 (41.7%)	10 (41.7%)	25 (49.0%)
Smoking status									
Current smoker	70 (17.5%)	1 (14.3%)	15 (13.3%)	7 (24.1%)	11 (21.6%)	1 (12.5%)	16 (13.9%)	6 (25.0%)	13 (25.5%)
Previous smoker	116 (29.1%)	2 (28.6%)	27 (23.9%)	10 (34.5%)	18 (35.5%)	2 (25.0%)	36 (31.3%)	9 (37.5%)	12 (23.5%)
Previous ischemic stroke	77 (19.3%)	1 (14.3%)	20 (17.7%)	5 (17.2%)	8 (15.7%)	3 (37.5%)	24 (20.9%)	2 (8.3%)	14 (27.5%)
Previous TIA	31 (7.8%)	0 (0.0%)	8 (7.1%)	1 (3.4%)	4 (7.8%)	1 (12.5%)	12 (10.4%)	1 (4.2%)	4 (7.8%)
Myocardial infarction	38 (9.5%)	1 (14.3%)	14 (12.4%)	3 (10.3%)	2 (3.9%)	1 (12.5%)	11 (9.6%)	1 (4.2%)	5 (9.8%)
Coronary artery disease	61 (15.3%)	1 (14.3%)	16 (14.2%)	6 (20.7%)	4 (7.8%)	2 (25.0%)	15 (13.0%)	6 (25.0%)	11 (21.6%)
TOAST classification									
Large artery sclerosis	7 (1.8%)	0 (0.0%)	4 (3.5%)	0 (0.0%)	2 (3.9%)	0 (0.0%)	1 (0.9%)	0 (0.0%)	0 (0.0%)
Cardioembolism	75 (18.8%)	0 (0.0%)	19 (14.8%)	10 (34.5%)	16 (31.4%)	0 (0.0%)	13 (11.3%)	5 (20.8%)	12 (23.5%)
Small vessel occlusion	118 (29.6%)	0 (0.0%)	35 (31.0%)	8 (27.6%)	12 (23.5%)	0 (0.0%)	40 (34.8%)	0 (0.0%)	17 (33.3%)
Stroke or other identified cause	1 (0.3%)	0 (0.0%)	0 (0.0%)	0 (0.0%)	0 (0.0%)	0 (0.0%)	1 (0.9%)	0 (0.0%)	0 (0.0%)
Stroke of unknown cause	197 (49.4%)	7 (100.0%)	55 (48.7%)	11 (37.9%)	21 (41.2%)	8 (100.0%)	60 (52.2%)	13 (54.2%)	22 (43.1%)
Score on NIHSS									
Median NIHSS (IQR)	3 (IQR 1–5)	2 (IQR 1–6)	3 (IQR 1–6)	3 (IQR 2–5)	3 (IQR 1–5)	3 (IQR 1–8)	2 (IQR 1–4)	2 (IQR 1–3)	3 (IQR 1–5)

### Clinical consequences of diagnostic procedures

1. Rhythm monitoring: As reported previously, AF was detected in 27 of 200 patients (13.5%) by enhanced and prolonged Holter-ECG monitoring in the intervention arm. [4] Twelve of 198 (6.1%) patients of the standard of care group had AF in routine ECGs. The older the patients were, the more often atrial fibrillation was detected (see Table 3). OAC was initiated in all patients. Four patients underwent pacemaker implantation within the follow-up period. None of them received it because of the Holter-ECG monitoring. The number needed to change decision (NNCD) was 8 for enhanced and prolonged monitoring (3x 10day Holter ECG, 95% CI = 5–12), 12 for the first 10-day Holter and 17 for usual care (95% CI = 10–33).
2. Echocardiography: We found 179 pathologies in 112 patients receiving echocardiography: 90 pathologies were diagnosed using TTE and 89 using TEE. There were two patients with five different pathologies, two with four pathologies, fifteen with three pathologies, 23 with two pathologies and 70 patients with only one cardiac pathology. We detected 44 patients with hypo- and/or akinesia of at least one myocardial segment (11.1%, 29 patients with only hypokinesia and 15 patients with hypo- and akinesia), 40 patients with patent foramen ovale (PFO) and / or atrial septal aneurysm (ASA) (10.1%, 7 patients with ASA only, 25 patients with PFO only and 8 patients with combined PFO and ASA), 34 patients with aortic plaques (8.5%, varying from 1 to 18 plaques per patient) and 27 patients with a reduced LVEF < 50% (6.8%, ranging from 15 to 49%). To reflect the age-dependency of pathologies detected by echocardiography, we divided the study population into three age groups: (I) < 70 years, n = 131, (II) 70–75 years, n = 132, and (III) > 75 years (n = 135). The younger the patients were the more often PFO and / or ASA was detected (p = 0.003), furthermore there were trends towards an age-dependency of aortic aneurysm (p = 0.061) and wall motion disorders (p = 0.094), see Table 3).

**Table 3:** Echocardiographic pathologies and potential therapeutic consequences.

pathology	Patients < 70 years (n = 131)	Patients 70–75 years (n = 132)	Patients > 75 years (n = 135)	P value^†^
Atrial fibrillation	10 (7.6%)	11 (8.3%)	15 (11.1%)	0.32
Detected by enhanced and prolonged Holter-ECG monitoring	8 (6.1%)	10 (7.6%)	9 (6.7)	0.86
Detected by usual care (Holter-) ECG monitoring	2 (1.5%)	1 (0.8%)	6 (4.4%)	0.11
LA thrombus (TEE)	0 (0%)	0 (0.0%)	0 (0.0%)	--
LAA thrombus (TEE)	1 (0.8%)	0 (0.0%)	0 (0.0%)	--
LV thrombus (TTE)	0 (0.0%)	0 (0.0%)	0 (0.0%)	--
LV aneurysm (TTE)	0 (0%)	0 (0.0%)	1 (0.7)	--
Myxoma (TEE)	1 (0.8%)	0 (0.0%)	0 (0.0%)	--
Endocarditis (TEE)	0 (0.0%)	0 (0.0%)	0 (0.0%)	--
PFO and/or ASA (TEE)	20 (15.3%)	14 (10.6%)	6 (4.4%)	0.003
Aortic aneurysm (TEE/TTE)	3 (2.3%)	1 (0.8%)	0 (0.0%)	0.061
Aortic plaques (TEE)	13 (9.9%)	13 (9.8%)	8 (5.9%)	0.24
Severe valve insufficiency (TTE)	0 (0%)	1 (0.8%)	0 (0.0%)	--
Severe valve stenosis (TTE)	0 (0%)	1 (0.8%)	1 (0.7%)	--
Valve thrombosis (TEE)	1 (0.8%)	1 (0.8%)	0 (0.0%)	--
LVEF < 50% (TTE)	9 (8.9%)	4 (3.0%)	14 (10.4%)	0.25
Hypo- and/or akinesia (TTE)	13 (9.9%)	9 (6.8%)	22 (16.3%)	0.094

^†^ p values are from Armitage's trend test

A high-risk source of cardiac thromboembolism was detected in four (1.0%) versus zero patients by TEE and TTE, respectively. A medium-risk source of cardioembolism was found in 40 patients (15.5%) by TEE, whereas none was detected by TTE.15 of all 383 patients who underwent echocardiography (3.9%), were treated as a consequence of the echocardiographic findings. Only one therapeutic decision was based on TTE. This corresponds to a rate of 0.3% of all performed 330 TTEs (Number needed to change decision (NNCD) 330) and to a rate of 9.0% of all conducted 155 TEEs (NNCD 12). In twelve patients, the therapy shifted from antiplatelet drugs to OAC, two patients underwent surgery and one patient received PFO closure. Most patients who received OAC were patients with PFO and/or ASA. Anticoagulation for PFO/ASA was mainly driven by centre policies (anticoagulation rates for PFO/ASA were 35.7%, 5.6%, 50.0%, 0.0% for centres 1–4, respectively). Therapy was shifted towards OAC because of deep vein thrombosis in two patients and because of repeated strokes of unknown cause in one case. Two patients had ASA only and were treated with OAC because of a right to left shunt documented with TEE or bubble test, respectively.

**Fig 3:**
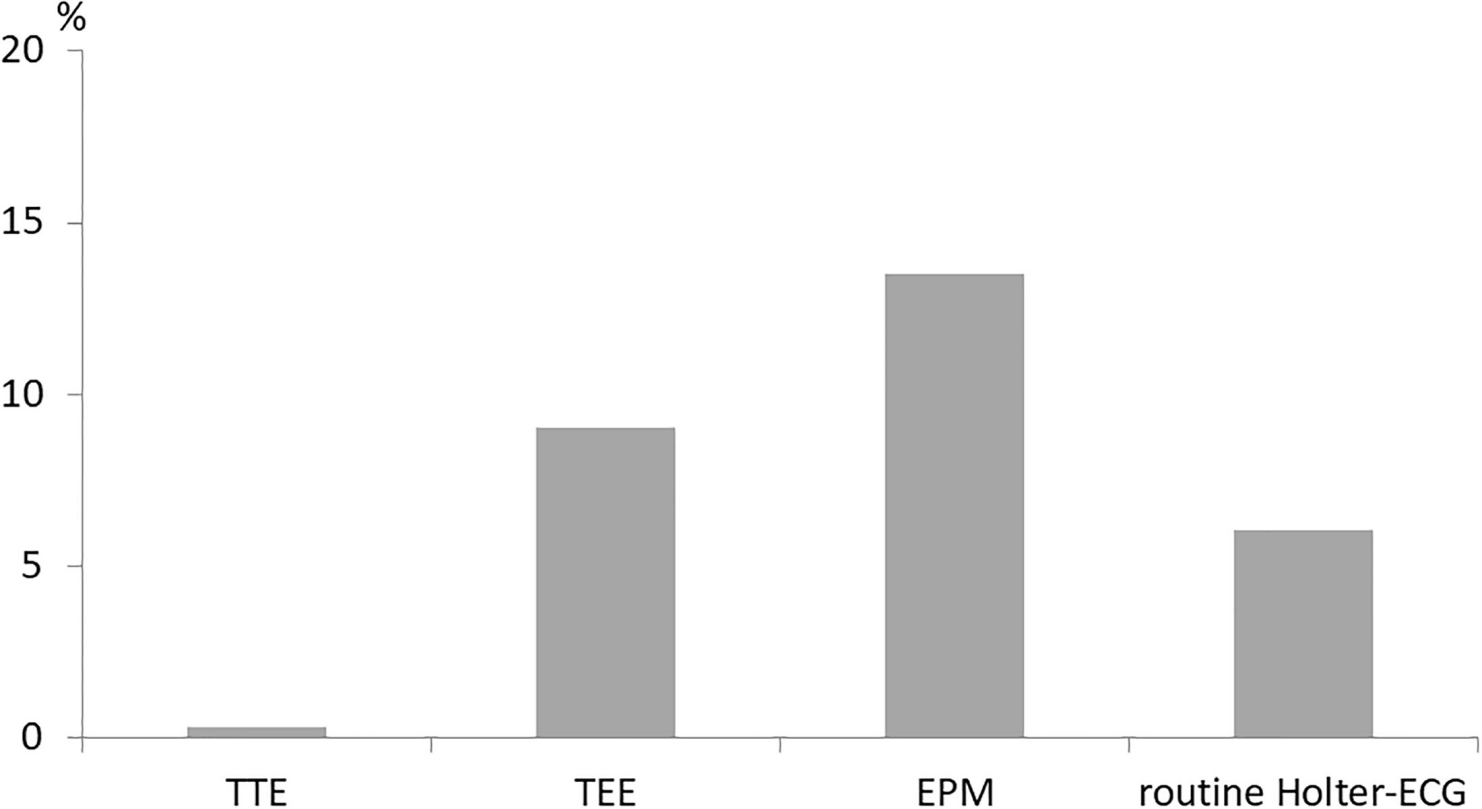
Rate of changes in therapeutic decisions based on echocardiography or ECG.

A comparison between the changes of therapeutic regime based on the four diagnostic methods (TTE, TEE, enhanced and prolonged Holter-ECG monitoring and routine (24-hours) ECG or telemetry) is shown in Fig 3.

### Prognosis of patients with pathologic ECG and echocardiography findings

**Fig 4:**
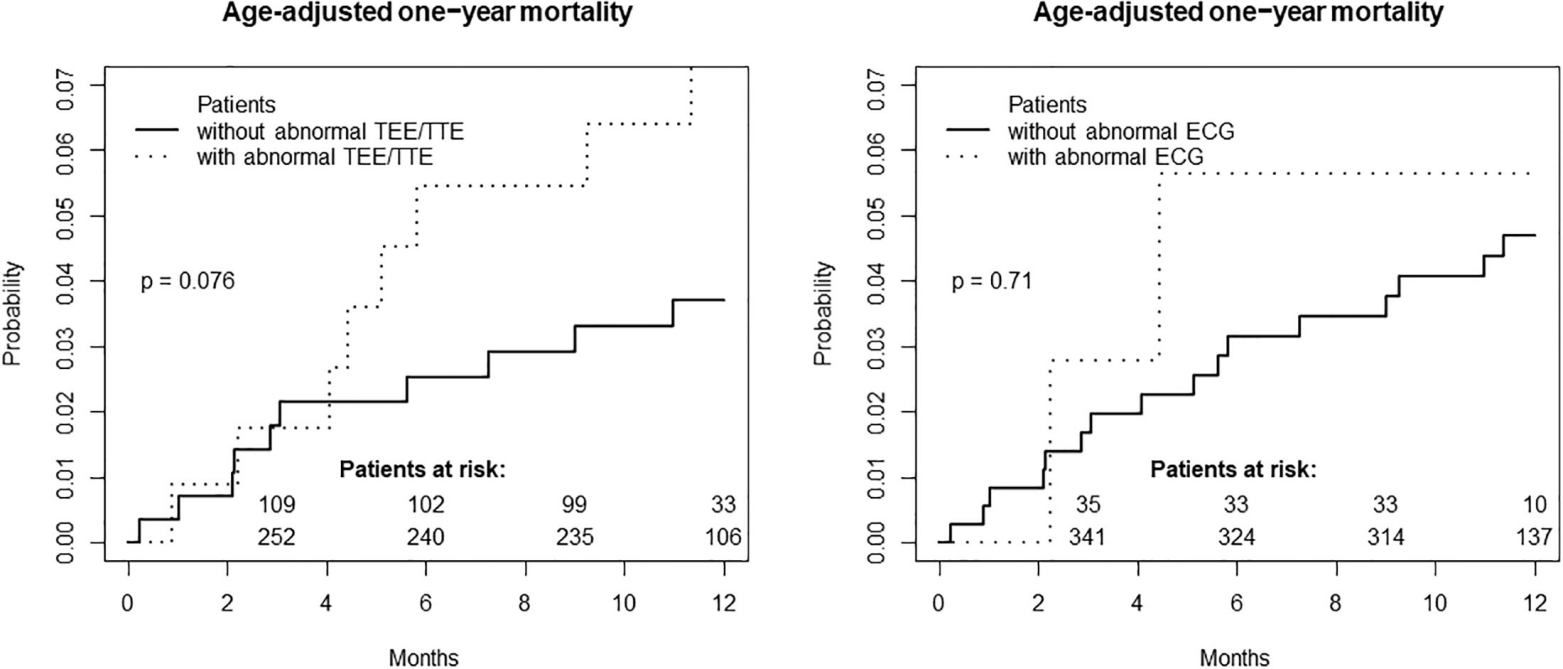
Age-adjusted one-year mortality in dependency of the presence of pathologic echocardiographic or ECG findings.

17 study patients died within one year. Of those, 16 received echocardiographic examinations. Three different echocardiographic pathologies were detected in these patients: wall motion abnormalities in six patients, a reduced LVEF and aortic plaques each in three patients. Patients with pathologic echocardiographic findings had a trend towards higher one-year mortality, whereas pathologic ECG findings were not associated with a higher age-adjusted one-year mortality (p = 0.076 vs. p = 0.71, see Fig 4). The death of all three patients with the combination of reduced LVEF and wall motion abnormalities was classified as “cardiovascular”.

## Discussion

We evaluated the diagnostic value of echocardiography in comparison to Holter-ECG monitoring in patients with acute ischemic stroke ≥ 60 years with respect to therapeutic consequences. While the study design allowed a randomised comparison between a strategy of prolonged ECG monitoring and usual care ECG monitoring, the analysis regarding echocardiography was based on observational non-randomised comparisons. Most therapeutic changes were based on repeated prolonged ECG-monitoring by means of three 10-day Holter-ECGs (13.5%). TEE and conventional monitoring led to a different therapy in 9.0% and 6.1%, respectively. TTE was responsible only for one new therapeutic decision (0.3%). Furthermore, our data showed a trend towards higher one-year mortality in patients with abnormal echocardiographic findings.

Previous studies have compared TEE and TTE. Similarly to our findings, the superiority of TEE over TTE in the diagnostics of cardiac sources of thromboembolism was also demonstrated in two previous studies that observed a change of therapeutic regimen in 41% or 21.4% of stroke patients because of TEE findings and 0% or 4.2% because of TTE findings [19,13]. The detection rate of high- or medium-risk sources of cardioembolism in our study (1.0% and 15.5%) was lower than previously reported (high-risk sources of cardioembolism in up to 10% of patients [20] and medium-risk sources in up to 59.4% of all investigated patients [13]). Counterintuitive, patients’ age does not seem to play an important role: whereas in a study with patients < 50 years found a potential source of cardiac embolism in 30% by TEE and in 10% by TTE [21], a study, which included only patients ≥ 80 years, documented a source of cardiac embolism in 35% of patients with TEE [22], which is higher in comparison to our data. Differences in definitions of high- and medium-risk sources of cardioembolism and pre-selection for TEE might explain the differences in detection rates of sources of cardiac embolism.

The most common reasons for therapeutic changes by echocardiography were PFO and ASA in combination (n = 5), isolated PFO (n = 3) or isolated ASA (n = 2). The most common therapeutic change was anticoagulation which is in line with previous trials. However, at the time of the study, therapeutic recommendations for PFO and ASA recommended anticoagulation in selected cases because three randomised trials CLOSURE-1, PC and RESPECT [23–25] were negative for PFO closure. Recently, the CLOSE and the REDUCE study clearly showed a benefit of PFO closure over medical management which will lead to a more prominent role of TEE in the near future [26–28] and more patients with PFO being treated by PFO closure. However, all patients in our study were aged 60 years and above whereas the patients in the randomised PFO trials were younger than 60 years. The detection of large PFOs by TTE may have had a major influence on the value of the procedure in terms of therapeutic consequences.

The most common diagnostic finding by prolonged monitoring was detection of AF and all patients with AF were treated with OAC according to current guidelines. Other diagnostic findings (e. g. AV block, pauses) were rare and did not lead to direct therapeutic consequences.

There was a trend towards a higher mortality rate in patients with echocardiographic pathologies. Wall motion abnormalities and a reduced LVEF were the most common pathologies which are indicators of heart failure and/or coronary artery disease. This may easily explain the increase in mortality risk. As a clinical consequence, the diagnostic workup in stroke patients should not be limited to the identification of the most likely aetiology of the occurred ischemic event, but consider the stroke patient as a patient at risk for other cardiovascular morbidities. Closer interdisciplinary cooperation between neurologists and cardiologists may have led to further diagnostic / therapeutic measures (e.g. cardiac catheterization, revascularization) and a prospective trial investigating a more invasive evaluation of these patients seems useful.

The one-year mortality did not differ significantly between the group of patients with and without pathologic ECG findings. This may be explained by the high rate of anticoagulation of detected AF cases.

The unique feature of our study is a head-to-head comparison of prolonged monitoring for 30 days in all patients in the intervention arm and echocardiographic data in 96% of the patients. This head-to-head comparison clearly shows that prolonged monitoring leads to 50% more therapeutic changes than TEE and 27fold more therapeutic changes than TTE. Hence, prolonged monitoring should receive a more prominent role in the workup of stroke patients in the future and a restriction to only 24 hours (as e.g. in the clinical construct of ESUS [29] or the guidelines of ESO or AHA/ASA [2,3]) cannot be recommended. Under conditions of cost pressure or limited resources, predictors of increased AF prevalence and therefore possible preselection criteria for prolonged ECG monitoring are age [30], left atrial size and function [31], enhanced supraventricular ectopy [32], elevated levels of natriuretic peptides [33]and others. In contrast, there is not much literature about preselection criteria for echocardiography.

Strengths of our study include the randomised, controlled and prospective multicenter study design and the four study centres representing rural, urban and teaching hospitals/university hospitals. However, echocardiography rates differed widely between the centres which limits generalisability and may reflect selection bias. The main reason for varying rates of TTE and TEE is local in-house practice of the four study centers, which represent two university, one large city and one hospital covering a rural area. It reflects different interpretations of the current guidelines. Importantly, the German Stroke Society requires at least 15% of patients with ischemic stroke to be investigated by transesophageal echocardiography. Furthermore, large and potentially relevant interatrial shunts can also be detected by TTE after application of a contrast agent. [34] Although performed in clinical practice, this approach was not sufficiently evaluated in clinical trials at that point and was not prospectively documented in our trial, but may lead to a more relevant role of TTE in the future.

One of the main limitations is our substudy design without a standardized algorithm and without a randomization of the two echocardiographic modalities, which would have been advantageous for the present question. Furthermore, our results were obtained in patients aged ≥ 60 years. The prevalence of PFO is likely to be higher in younger stroke patients [35], whereas the prevalence of AF is supposed to be higher in older patients [30], which might partly explain the diagnostic superiority of repeated and prolonged Holter-ECG monitoring in our study cohort. Additionally, although Find-AF_RANDOMISED_ was a prospective randomised trial, this analysis was not pre-specified and should therefore be considered descriptive and hypothesis-generating.

In summary, our results show that enhanced and prolonged ECG-monitoring is the key tool in the cardiac workup of ischemic stroke in patients older than 60 years and should therefore play a more prominent role in future guidelines. Echocardiography of any type helps to identify a subgroup of stroke patients at high cardiovascular risk. A better neurocardiologic diagnostic and therapeutic collaboration and further appropriate trials are warranted.

## Supporting information

S1 Fig Find-AF_RANDOMISED_ study protocol. (PDF)

S1 Table CONSORT checklist. (DOC)

S2 Table Raw data Find-AF_RANDOMISED_ study subanalysis. (XLSX)

## References

[1] Burn J, Dennis M, Bamford J, Sandercock P, Wade D, Warlow C (1994) Long-term risk of recurrent stroke after a first-ever stroke. The Oxfordshire Community Stroke Project. Stroke 25 (2): 333–337. 8303740 10.1161/01.str.25.2.333

[2] European Stroke Organisation (ESO) Executive Committee EWC (2008) Guidelines for management of ischaemic stroke and transient ischaemic attack 2008. Cerebrovascular diseases (Basel, Switzerland) 25 (5): 457–507. 10.1159/000131083 18477843

[3] Jauch EC, Saver JL, Adams HP, Bruno A, Connors JJB, Demaerschalk BM et al (2013) Guidelines for the early management of patients with acute ischemic stroke. A guideline for healthcare professionals from the American Heart Association/American Stroke Association. Stroke 44 (3): 870–947. 10.1161/STR.0b013e318284056a 23370205

[4] Wachter R, Gröschel K, Gelbrich G, Hamann GF, Kermer P, Liman J et al (2017) Holter-electrocardiogram-monitoring in patients with acute ischaemic stroke (Find-AFRANDOMISED). An open-label randomised controlled trial. The Lancet. Neurology 16 (4): 282–290. 10.1016/S1474-4422(17)30002-9 28187920

[5] Grond M, Jauss M, Hamann G, Stark E, Veltkamp R, Nabavi D et al (2013) Improved detection of silent atrial fibrillation using 72-hour Holter ECG in patients with ischemic stroke. A prospective multicenter cohort study. Stroke 44 (12): 3357–3364. 10.1161/STROKEAHA.113.001884 24130137

[6] Stahrenberg R, Weber-Krüger M, Seegers J, Edelmann F, Lahno R, Haase B et al (2010) Enhanced detection of paroxysmal atrial fibrillation by early and prolonged continuous holter monitoring in patients with cerebral ischemia presenting in sinus rhythm. Stroke 41 (12): 2884–2888. 10.1161/STROKEAHA.110.591958 20966415

[7] Hart RG, Pearce LA, Aguilar MI (2007) Meta-analysis. Antithrombotic therapy to prevent stroke in patients who have nonvalvular atrial fibrillation. Annals of internal medicine 146 (12): 857–867. 17577005 10.7326/0003-4819-146-12-200706190-00007

[8] Kirchhof P, Benussi S, Kotecha D, Ahlsson A, Atar D, Casadei B et al (2016) 2016 ESC Guidelines for the management of atrial fibrillation developed in collaboration with EACTS. European heart journal 37 (38): 2893–2962. 10.1093/eurheartj/ehw210 27567408

[9] Häusler K, Gröschel K, Köhrmann M, Schnabel R, Anker S, Brachmann J et al (2017) Positionspapier zur Detektion von Vorhofflimmern nach ischämischem Schlaganfall. Akt Neurol.

[10] Adams HP, del Zoppo G, Alberts MJ, Bhatt DL, Brass L, Furlan A et al (2007) Guidelines for the early management of adults with ischemic stroke. A guideline from the American Heart Association/American Stroke Association Stroke Council, Clinical Cardiology Council, Cardiovascular Radiology and Intervention Council, and the Atherosclerotic Peripheral Vascular Disease and Quality of Care Outcomes in Research Interdisciplinary Working Groups: The American Academy of Neurology affirms the value of this guideline as an educational tool for neurologists. Circulation 115 (20): e478–534. 10.1161/CIRCULATIONAHA.107.181486 17515473

[11] Ringelstein EB, Chamorro A, Kaste M, Langhorne P, Leys D, Lyrer P et al (2013) European Stroke Organisation recommendations to establish a stroke unit and stroke center. Stroke 44 (3): 828–840. 10.1161/STROKEAHA.112.670430 23362084

[12] Kapral MK, Silver FL (1999) Preventive health care, 1999 update. 2. Echocardiography for the detection of a cardiac source of embolus in patients with stroke. Canadian Task Force on Preventive Health Care. CMAJ: Canadian Medical Association journal = journal de l'Association medicale canadienne 161 (8): 989–996. 10551199 PMC1230713

[13] de Bruijn SFTM, Agema WRP, Lammers GJ, van der Wall EE, Wolterbeek R, Holman ER et al (2006) Transesophageal echocardiography is superior to transthoracic echocardiography in management of patients of any age with transient ischemic attack or stroke. Stroke 37 (10): 2531–2534. 10.1161/01.STR.0000241064.46659.69 16946152

[14] Lerakis S, Nicholson WJ (2005) Part I. Use of echocardiography in the evaluation of patients with suspected cardioembolic stroke. The American journal of the medical sciences 329 (6): 310–316. 15958873 10.1097/00000441-200506000-00011

[15] http://www.dsg-info.de/stroke-units/zertifizierungsantraege--zertifizierungskriterien.html DSG Internetseite.

[16] Weber-Krüger M, Gelbrich G, Stahrenberg R, Liman J, Kermer P, Hamann GF et al (2014) Finding atrial fibrillation in stroke patients. Randomized evaluation of enhanced and prolonged Holter monitoring—Find-AF(RANDOMISED)—rationale and design. American heart journal 168 (4): 438–445.e1. 10.1016/j.ahj.2014.06.018 25262252

[17] Saric M, Armour AC, Arnaout MS, Chaudhry FA, Grimm RA, Kronzon I et al (2016) Guidelines for the Use of Echocardiography in the Evaluation of a Cardiac Source of Embolism. Journal of the American Society of Echocardiography: official publication of the American Society of Echocardiography 29 (1): 1–42. 26765302 10.1016/j.echo.2015.09.011

[18] Adams HP, Bendixen BH, Kappelle LJ, Biller J, Love BB, Gordon DL et al (1993) Classification of subtype of acute ischemic stroke. Definitions for use in a multicenter clinical trial. TOAST. Trial of Org 10172 in Acute Stroke Treatment. Stroke 24 (1): 35–41. 7678184 10.1161/01.str.24.1.35

[19] Blum A, Reisner S, Farbstein Y (2004) Transesophageal echocardiography (TEE) vs. transthoracic echocardiography (TTE) in assessing cardio-vascular sources of emboli in patients with acute ischemic stroke. Medical science monitor: international medical journal of experimental and clinical research 10 (9): CR521–3. 15328485

[20] Strandberg M, Marttila RJ, Helenius H, Hartiala J (2002) Transoesophageal echocardiography in selecting patients for anticoagulation after ischaemic stroke or transient ischaemic attack. Journal of neurology, neurosurgery, and psychiatry 73 (1): 29–33. 10.1136/jnnp.73.1.29 12082041 PMC1757302

[21] Rettig TCD, Bouma BJ, van den Brink RBA (2009) Influence of transoesophageal echocardiography on therapy and prognosis in young patients with TIA or ischaemic stroke. Netherlands heart journal: monthly journal of the Netherlands Society of Cardiology and the Netherlands Heart Foundation 17 (10): 373–377. 10.1007/BF03086287 PMC2773028 19949646

[22] Nakanishi K, Homma S (2016) Role of echocardiography in patients with stroke. Journal of cardiology 68 (2): 91–99. 10.1016/j.jjcc.2016.05.001 27256218

[23] Furlan AJ, Reisman M, Massaro J, Mauri L, Adams H, Albers GW et al (2012) Closure or medical therapy for cryptogenic stroke with patent foramen ovale. The New England journal of medicine 366 (11): 991–999. 10.1056/NEJMoa1009639 22417252

[24] Carroll JD, Saver JL, Thaler DE, Smalling RW, Berry S, MacDonald LA et al (2013) Closure of Patent Foramen Ovale versus Medical Therapy after Cryptogenic Stroke. The New England journal of medicine 368 (12): 1092–1100. 10.1056/NEJMoa1301440 23514286

[25] Meier B, Kalesan B, Mattle HP, Khattab AA, Hildick-Smith D, Dudek D et al (2013) Percutaneous closure of patent foramen ovale in cryptogenic embolism. The New England journal of medicine 368 (12): 1083–1091. 10.1056/NEJMoa1211716 23514285

[26] Mas J-L, Derumeaux G, Guillon B, Massardier E, Hosseini H, Mechtouff L et al (2017) Patent Foramen Ovale Closure or Anticoagulation vs. Antiplatelets after Stroke. The New England journal of medicine 377 (11): 1011–1021. 10.1056/NEJMoa1705915 28902593

[27] Saver JL, Carroll JD, Thaler DE, Smalling RW, MacDonald LA, Marks DS et al (2017) Long-Term Outcomes of Patent Foramen Ovale Closure or Medical Therapy after Stroke. The New England journal of medicine 377 (11): 1022–1032. 10.1056/NEJMoa1610057 28902590

[28] Søndergaard L, Kasner SE, Rhodes JF, Andersen G, Iversen HK, Nielsen-Kudsk JE et al (2017) Patent Foramen Ovale Closure or Antiplatelet Therapy for Cryptogenic Stroke. The New England journal of medicine 377 (11): 1033–1042. 10.1056/NEJMoa1707404 28902580

[29] Hart RG, Diener H-C, Coutts SB, Easton JD, Granger CB, O'Donnell MJ et al (2014) Embolic strokes of undetermined source. The case for a new clinical construct. The Lancet. Neurology 13 (4): 429–438. 10.1016/S1474-4422(13)70310-7 24646875

[30] Wachter R, Weber-Krüger M, Seegers J, Edelmann F, Wohlfahrt J, Wasser K et al (2013) Age-dependent yield of screening for undetected atrial fibrillation in stroke patients. The Find-AF study. Journal of neurology 260 (8): 2042–2045. 10.1007/s00415-013-6935-x 23632947 PMC3734596

[31] Stahrenberg R, Edelmann F, Haase B, Lahno R, Seegers J, Weber-Krüger M et al (2011) Transthoracic echocardiography to rule out paroxysmal atrial fibrillation as a cause of stroke or transient ischemic attack. Stroke 42 (12): 3643–3645. 10.1161/STROKEAHA.111.632836 21998056

[32] Dewland TA, Vittinghoff E, Mandyam MC, Heckbert SR, Siscovick DS, Stein PK et al (2013) Atrial ectopy as a predictor of incident atrial fibrillation. A cohort study. Annals of internal medicine 159 (11): 721–728. 10.7326/0003-4819-159-11-201312030-00004 24297188 PMC4115459

[33] Wachter R, Lahno R, Haase B, Weber-Krüger M, Seegers J, Edelmann F et al (2012) Natriuretic peptides for the detection of paroxysmal atrial fibrillation in patients with cerebral ischemia—the Find-AF study. PloS one 7 (4): e34351 10.1371/journal.pone.0034351 22509292 PMC3324530

[34] Zhao E, Wei Y, Zhang Y, Zhai N, Zhao P, Liu B (2015) A Comparison of Transthroracic Echocardiograpy and Transcranial Doppler With Contrast Agent for Detection of Patent Foramen Ovale With or Without the Valsalva Maneuver. Medicine 94 (43): e1937 10.1097/MD.0000000000001937 26512622 PMC4985435

[35] Gupta V, Yesilbursa D, Huang WY, Aggarwal K, Gupta V, Gomez C et al (2008) Patent foramen ovale in a large population of ischemic stroke patients. Diagnosis, age distribution, gender, and race. Echocardiography (Mount Kisco, N.Y.) 25 (2): 217–227. 10.1111/j.1540-8175.2007.00583.x 18269568

