# Supplementary material for: The cardiac diagnostic work-up in stroke patients—A subanalysis of the Find-AFRANDOMISED trial
Source: PLoS One. 2019 May 9;14(5):e0216530. doi: 10.1371/journal.pone.0216530 (PMC6508702; doi:10.1371/journal.pone.0216530)
Supplement: S1 Fig — (PDF) [file pone.0216530.s001.pdf]

Finding Atrial Fibrillation in stroke – randomised evaluation of enhanced and prolonged Holter monitoring

A prospective, randomised, controlled study to determine the detection of atrial fibrillation by prolonged and enhanced Holter monitoring as compared to usual care in stroke patients

(Find-AF$_{RANDOMISED}$)

ISRCTN: will be provided after approval by the responsible ethics committee

Coordinating investigators

PD Dr. med. Rolf Wachter
Department of Cardiology and Pneumology
University of Göttingen
Robert-Koch-Str. 40
37075 Göttingen, Germany
Phone: +49-551-39-9258
Fax: +49-551-39-22600
e-mail: wachter@med.uni-goettingen.de

PD Dr. med. Klaus Gröschel
Clinic and Policlinic for Neurology
University of Mainz
Langenbeckstraße 1
55131 Mainz, Germany
Phone: +49-6131-173105
Fax: +49-6131-17-47-3105
e-mail: Klaus.groeschel@unimedizin-mainz.de

Biometry

Prof. Dr. rer. nat. Dr. rer. med. Götz Gelbrich
Department for Epidemiology and Clinical Biometry
University of Würzburg
Würzburg, Germany
Phone: +49-931- 20147312
Fax: +49-931-201 647310
e-mail: goetz.gelbrich@klinik.uni-wuerzburg.de

Sponsor

Universitätsmedizin Göttingen
Bereich Humanmedizin
Robert-Koch-Str. 40
37075 Göttingen, Germany

Study protocol Version 3.0, 10.07.2014

The information provided in this document is confidential and is not to be circulated to third parties.

TABLE OF CONTENTS

PAGE

GENERAL INFORMATION 4

Study team 4
Synopsis 7
Flow Chart 15
Evaluation and visit schedule 16

1 RATIONALE 17

1.1 Medical background 17
1.2 Rationale 17
1.3 Risk-Benefit Consideration 18

2 OBJECTIVES 20

2.1 Primary objectives 20
2.2 Secondary objectives 20

3 TRIAL DESIGN AND DESCRIPTION 21

3.1 Trial design 21
3.2 Requirements for participating investigators and trial sites 21
3.3 Trial sites and number of trial subjects 21
3.4 Expected duration of trial 21
3.5 Premature termination 22

4 SELECTION OF TRIAL SUBJECTS 23

4.1 Inclusion criteria 23
4.2 Exclusion criteria 23
4.3 Gender aspects 23

5 TRIAL INTERVENTIONS 24

5.1 Usual care (basic therapy) 24
5.2 Enhanced and prolonged Holter monitoring 24

6 INDIVIDUAL TRIAL PROCEDURES 27

6.1 Screening 27
6.2 Patient information and informed consent 28
6.3 Enrolment and randomisation (V0) 29
6.4 Trial visits 30
6.5 Processing and storage of blood samples, evaluation of Biomarkers 32
6.6 Premature termination of trial participation 32

7 ADVERSE EVENTS AND SERIOUS ADVERSE EVENTS (AE/SAE) 34

7.1 Definitions 34
7.2 Documentation and Reporting 35

8 BIOMETRICAL ASPECTS 36

8.1 Randomisation algorithm 36
8.2 Endpoints 36
8.3 Statistical hypothesis 39
8.4 Sample Size Discussion 39
8.5 Statistical methods 40
8.6 Interim Analysis 40
8.7 Final Analysis 40

9 ETHICAL, LEGAL AND ADMINISTRATIVE ASPECTS 41
9.1 Submission 41
9.2 Protocol Amendments 41
10 DATA HANDLING AND RECORD KEEPING 42
10.1 Case Report Forms (CRF) 42
10.2 Data Management 43
10.3 Archival storage 43
11 REFERENCE EVALUATIONS 45
12 SUPERVISION OF CLINICAL TRIAL 46
12.1 Direct access to source data 46
12.2 Monitoring 46
12.3 Audits 47
13 DATA PROTECTION 48
14 ADMINISTRATIVE AGREEMENTS 49
14.1 Adherence to the protocol 49
14.2 Funding and Insurance 49
14.3 Publication policy 49
15 REFERENCES GENERAL 51
16 CONFIRMATION OF THE FINAL PROTOCOL 52
17 PROTOCOL AGREEMENT 53
17.1 References related to Find-AF$_{RANDOMISED}$ 54
18 APPENDIX 56

GENERAL INFORMATION

Study team

**Sponsor**	Universitätsmedizin Göttingen<br>Robert-Koch-Str. 40<br>37075 Göttingen
**Coordinating investigators**	*PD Dr. med. Rolf Wachter*<br>Department of Cardiology and Pneumology<br>University of Göttingen<br>Robert-Koch-Str. 40<br>37075 Göttingen, Germany<br>Phone: +49-551-39-9258<br>Fax: +49-551-39-22600<br>e-mail: wachter@med.uni-goettingen.de<br><br>*PD Dr. med. Klaus Gröschel*<br>Clinic and Policlinic for Neurology<br>University of Mainz<br>Langenbeckstraße 1<br>55131 Mainz, Germany<br>Phone: +49-6131-173105<br>Fax: +49-6131-17-47-3105<br>e-mail: Klaus.groeschel@unimedizin-mainz.de
**Study Coordination**	*Mark Weber-Krüger*<br>Department of Cardiology and Pneumology<br>University of Göttingen<br>Robert-Koch-Str. 40<br>37075 Göttingen, Germany<br>Phone: +49-551-39-8591<br>Fax: +49-551-39-22600<br>e-mail: mark.weber-krueger@med.uni-goettingen.de

**Biometry**	*Prof. Dr. rer. nat. Dr. rer. med. Götz Gelbrich*<br>Department of Epidemiology und Clinical Biometry<br>University of Würzburg<br>Würzburg, Germany<br>Phone: +49-931- 20147312<br>Fax: +49-931-201 647310<br>e-mail: goetz.gelbrich@klinik.uni-wuerzburg.de
**Study Project and Data Management**	Institut für anwendungsorientierte Forschung und klinische Studien<br>Universitätsmedizin Göttingen<br>Von-Bar-Str.2/4<br>37075 Göttingen
**Monitoring**	Institut für anwendungsorientierte Forschung und klinische Studien<br>Universitätsmedizin Göttingen<br>Von-Bar-Str.2/4<br>37075 Göttingen
**Reference Laboratory Holter ECG**	*Dr. med. Joachim Seegers*<br>Department of Cardiology and Pneumology<br>University of Göttingen<br>Robert-Koch-Str. 40<br>37075 Göttingen, Germany<br>Phone: +49-551-39-8591<br>Fax: +49-551-39- 19127<br>e-mail: jseegers@med.uni-goettingen.de
**Funding**	*Dr. Miriam Bach*<br>Boehringer Ingelheim Pharma GmbH & Co. KG<br>Regional MD Western Europe<br>Tel.: +49 (6132) 77-141123<br>miriam.bach@boehringer-ingelheim.com

**Atrial Fibrillation Endpoint Adjudication Committee**	*Chairman: Prof. Dr. med. David Conen* (Basel); Members: *Prof. Dr. med. Markus Zabel* (Göttingen), *Prof. Dr. med. Ulrich Laufs* (Homburg/Saar)
**Stroke Subtype Adjudication Committee**	*Chairman: Prof. Dr. med. Peter Heuschmann (Würzburg); Members: Dr. med. Eric Jüttler (Ulm), PD Dr. med. Holger Poppert (Munich)*

Synopsis

**APPLICANT/ COORDINATING INVESTIGATOR**	Applicant:<br>PD Dr. med. Rolf Wachter<br>Department of Cardiology and Pneumology<br>University of Göttingen<br>Robert-Koch-Str. 40<br>37075 Göttingen, Germany<br>e-mail: wachter@med.uni-goettingen.de<br><br>Co-Applicant:<br>PD Dr. med. Klaus Gröschel<br>Clinic and Policlinic for Neurology<br>University of Mainz<br>Langenbeckstraße 1<br>55131 Mainz, Germany<br>e-mail: klaus.groeschel@unimedizin-mainz.de
**TITLE OF STUDY**	Finding Atrial Fibrillation in stroke – randomised evaluation of enhanced and prolonged Holter monitoring
**CONDITION**	Stroke, secondary prevention
**OBJECTIVE(S)**	Primary Objectives: To assess whether repeated enhanced and prolonged ECG monitoring after ischemic stroke results in a higher detection of atrial fibrillation (/flutter) compared to usual care (at least 24 hour of cardiac monitoring)<br>Secondary Objectives:<br>- Predictors of underlying paroxysmal atrial fibrillation<br>- Defining the ideal duration and timing of extended monitoring<br>- Feasibility of enhanced and prolonged procedures

**INTERVENTION(S)**	**<u>Experimental procedures</u>:**<br>Prolonged ECG monitoring (10-day Holter ECG at months 0, 3 and 6)<br>**<u>Control procedures</u>:**<br>Usual care according to current guidelines (minimum of 24 hours of cardiac monitoring)<br>**<u>Follow-up per patient</u>:**<br>12 months for the randomised trials, 24 months of additional observation for endpoints months<br>**<u>Duration of procedures per patient</u>:**<br>6 months (10-day Holter ECG at months 0, 3 and 6)
**INCLUSION AND EXCLUSION CRITERIA**	**<u>Inclusion criteria</u>:**<br>1. Recent cerebral ischemia defined as stroke (sudden focal neurologic deficit lasting > 24h consistent with the territory of a major cerebral artery and categorised as ischemic) and/or a corresponding lesion on brain imaging.<br>2. Stroke symptoms started ≤ 7 days ago.<br>3. Age ≥ 60 years.<br>4. Modified Rankin scale ≤ 2 (prior to index event).<br>**<u>Exclusion criteria</u>:**<br>1. Known history of atrial fibrillation/flutter or atrial fibrillation/flutter on admission ECG.<br>2. Indication for oral anticoagulation at randomisation.<br>3. Absolute contra-indication against oral anticoagulation at randomisation.<br>4. Intracerebral bleeding in medical history.<br>5. Patient scheduled for Holter-ECG or cardiac Event-Recording monitoring ≥ 48 hours.<br>6. Significant carotid artery or vertebral artery stenosis > 50% (NASCET classification), significant intracranial artery stenosis suspicious of atherosclerotic origin or acute arterial dissection explanatory of stroke symptoms.<br>7. Implanted pacemaker device or cardioverter/defibrillator.

	8. Life expectancy <1 year for reasons other than stroke (e.g. metastatic cancer).<br>9. Concomitant participation in another controlled randomised trial.
**OUTCOME(S)**	**<u>Primary efficacy endpoint</u>:**<br>The primary endpoint is the detection of newly diagnosed atrial fibrillation/flutter within 6 months and before occurrence of recurrent stroke or systemic embolism.<br>**<u>Secondary endpoints</u>:**<br>**Major secondary endpoints**<br>1. Detection of newly diagnosed atrial fibrillation (/flutter) as defined for the primary endpoint but within 12 months.<br>2. Detection of newly diagnosed atrial fibrillation (/flutter) as defined for the primary endpoint with the exception that hospitalisation for atrial fibrillation (/flutter) will be considered as censoring.<br>3. Recurrent stroke or systemic embolism after 12 months.<br>4. Total death after 12 months.<br>**Other secondary endpoints (all after 12 months)**<br>1. Cardiovascular death<br>2. Cerebrovascular death<br>3. Transient ischemic attack<br>4. Myocardial infarction<br>5. Bleeding complications<br>6. Quality of Life<br>7. Incremental detection of atrial fibrillation (/flutter) in the extended Holter monitoring periods after 3 and 6 months<br>8. Costs<br>9. Feasibility of monitoring procedures<br>**<u>Assessment of safety:</u>**<br>Procedures applied do not bear significant risks.
**STUDY TYPE**	Secondary prevention trial

**STATISTICAL ANALYSIS**	Efficacy:<br>Comparison of detection of atrial fibrillation (/ flutter) within 6 months<br>Description of the primary efficacy analysis and population:<br>Kaplan-Meier estimates with logrank-test, intention-to-treat<br>Safety:<br>No significant harm of the procedure.<br>Secondary endpoint(s):<br>Stroke, systemic embolism.
**SAMPLE SIZE**	To be assessed for eligibility: **(n = 1000)**<br>To be allocated to trial: **(n = 400)**<br>To be analysed: **(n =340)**
**TRIAL DURATION**	First patient in to last patient out (months): 24<br>Duration of the entire trial (months): 30<br>Recruitment period (months): 12
**PARTICIPATING CENTERS**	**n = 4**

Zusammenfassung

**ANTRAGSSTELLER /WISSENSCHAFT-LICHE LEITUNG**	Antragssteller:<br>PD Dr. med. Rolf Wachter<br>Abteilung für Kardiologie und Pneumologie<br>Universitätsmedizin Göttingen<br>Robert-Koch-Str. 40<br>37075 Göttingen, Germany<br>e-mail: wachter@med.uni-goettingen.de<br><br>Stellvertretender Antragssteller:<br>PD Dr. med. Klaus Gröschel<br>Klinik und Poliklinik für Neurologie<br>Universitätsmedizin Mainz<br>Langenbeckstraße 1<br>55131 Mainz, Germany<br>e-mail: klaus.groeschel@unimedizin-mainz.de
**TITEL DER STUDIE**	Detektion von Vorhofflimmern nach Schlaganfall – randomisierte Evaluation von verbessertem und prolongiertem Langzeit-EKG-Monitoring
**KRANKHEITSBILD**	Schlaganfall, Sekundärprävention
**ZIEL(E)**	Evaluation, ob ein wiederholtes prolongiertes und intensiviertes EKG-Monitoring nach ischämischem Schlaganfall im Vergleich zum diagnostischen Standard (kardiales Monitoring über minimal 24-Stunden) zu einer höheren Detektion von Vorhofflimmern (/-flattern) führt.

**INTERVENTIONEN**	<u>Prozeduren in der Untersuchungsgruppe</u>:<br>Prolongiertes EKG-Monitoring (10-Tage-Langzeit-EKG) bei Aufnahme, sowie nach 3 und 6 Monaten<br><u>Prozeduren in der Kontrollgruppe</u>:<br>Diagnostischer Standard (leitliniengerecht mindestens 24-Stunden EKG-Monitoring)<br><br><u>Follow-up pro Patient</u>:<br>12 Monate 24 Monate Nachbeobachtung nach der randomisierten Studie<br><u>Dauer der Prozeduren pro Patient</u>:<br>6 Monate (10-Tage-Langzeit-EKG nach 0, 3 und 6 Monaten)
**EIN-/AUSSCHLUSS-KRITERIEN**	<u>Einschluss-Kriterien</u>:<br>1. Diagnose eines akuten ischämischen Schlaganfalls (plötzliches fokales neurologisches Defizit über > 24h entsprechend eines Versorgungsgebietes einer großen hirnversorgenden Arterie und Klassifikation als ischämisches Ereignis) und/oder korrespondierende Läsion in der zerebralen Bildgebung.<br>2. Schlaganfall-assoziierte Symptome seit ≤ 7 Tagen<br>3. Alter ≥ 60 Jahre.<br>4. Modified Rankin Scale ≤ 2 (vor dem Indexereignis)<br><u>Ausschlusskriterien</u>:<br>1. Vorhofflimmern / Vorhofflattern vorbekannt oder im Aufnahme-EKG<br>2. Indikation zur oralen Antikoagulation vor Randomisierung.<br>3. Absolute Kontraindikation gegen orale Antikoagulation vor Randomisierung.<br>4. Intrazerebrale Blutung in der Vorgeschichte.<br>5. Langzeit-EKG/Event-Recorder > 48h (studienunabhängig) angesetzt.<br>6. Signifikante Arteria-carotis-interna- oder Arteria-vertebralis-Stenose (NASCET > 50%), signifikante intrakranielle Stenose mit Verdacht auf atherosklerotische Genese oder akute arterielle Dissektion als Erklärung der aufgetretenen Beschwerden.

	7. Implantierter Herzschrittmacher/Cardioverter-Defibrillator.<br>8. Lebenserwartung < 1 Jahr (z.B. aufgrund metastasierten Krebsleidens).<br>9. Teilnahme an einer anderen kontrollierten randomisierten Studie.
**ENDPUNKTE**	Primärer Wirksamkeits-Endpunkt<br>Detektion von Vorhofflimmern (-flattern) innerhalb von 6 Monaten<br><br>Sekundäre Endpunkte:<br>Hauptsächliche sekundäre Endpunkte:<br>1. Detektion von Vorhofflimmern (/-flattern) (siehe primärer Endpunkt), aber nach 12 Monaten.<br>2. Detektion von Vorhofflimmern (siehe primärer Endpunkt), jedoch mit der Ausnahme, dass Hospitalisation aufgrund von Vorhofflimmern (/-flattern) als zensierendes Ereignis betrachtet wird.<br>3. Schlaganfall-Rezidiv oder systemische Embolie nach 12 Monaten.<br>4. Todesfälle nach 12 Monaten.<br>Andere sekundäre Endpunkte (alle nach 12 Monaten):<br>1. Kardiovaskulärer Tod<br>2. Zerebrovaskulärer Tod<br>3. Transitorisch ischämische Attacke<br>4. Herzinfarkt<br>5. Blutungskomplikationen<br>6. Lebensqualität<br>7. Inkrementelle Detektion von Vorhofflimmern (/-flattern) in den prolongierten Monitoring-Episoden nach 3 und 6 Monaten<br>8. Kosten<br>9. Durchführbarkeit des prolongierten Monitoring-Verfahrens<br>Sicherheitsbestimmungen:<br>Es werden keine relevanten Risiken durch die Intervention erwartet.
**STUDIENTYP**	Sekundärpräventions-Studie

**STATISTISCHE ANALYSEN**	Wirksamkeit:<br>Vergleich der Detektion von Vorhofflimmern (/-flattern) nach 6 Monaten<br>Beschreibung der primären Wirksamkeitsanalysen und der Population:<br>Kaplan-Meier-Schätzungen mit Logrank-Test, Intention-to-treat<br>Sicherheit:<br>Keine relevanten Sicherheitsbedenken durch die durchgeführte Intervention.<br>Sekundäre(r) Endpunkt(e):<br>Schlaganfall, systemische Embolie
**STUDIENGRÖßE**	Zur Eignung geprüft: **(n = 1000)**<br>Zur Teilnahme an der Studie: **(n = 400)**<br>Zur Analyse: **(n =340)**
**STUDIENDAUER**	Erster Patient eingeschlossen bis letzter Patient absolviert (Monate): 24<br>Dauer der gesamten Studie (Monate): 30<br>Rekrutierungsphase (Monate): 12
**TEILNEHMENDE ZENTREN**	**n = 4**

Flow Chart

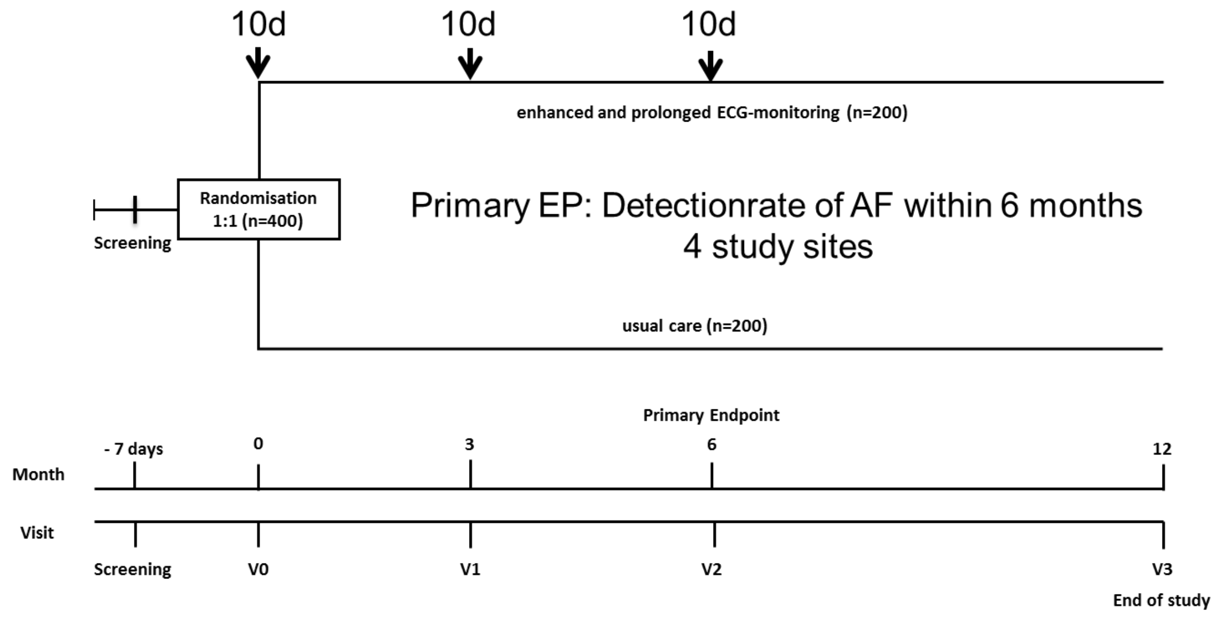
10d
10d
10d
enhanced and prolonged ECG-monitoring (n=200)
Randomisation
1:1 (n=400)
Screening
Primary EP: Detectionrate of AF within 6 months
4 study sites
usual care (n=200)
Primary Endpoint
Month
- 7 days
0
3
6
12
Visit
Screening
V0
V1
V2
V3
End of study

Evaluation and visit schedule

visit	scr/ V0	V1	V2	V3	V4	V5	V6	V7
time (days) ± (days)	0	90 ± 14	180 ± 14	360 ± 30	540 ± 30	720 ± 30	900 ± 30	1080 ± 30
study visit	🏠	🏠	🏠	🏠	☎	☎	☎	☎
inclusion/exclusion criteria	X							
written informed consent	X							
randomisation	X							
medical history	X							
morbidity/mortality/EP	X	X	X	X	X	X	X	X
concomitant medication	X	X	X	X	X	X	X	X
adverse events	X	X	X	X	X	X	X	X
guideline adherence	X	X	X	X	X	X	X	X
vital signs	X	X	X	X				
NIHSS	X	X	X	X				
mRS	X	X	X	X	X	X	X	X
quality of life	X	X		X				
healthcare utilisation	X		X	X				
10d-Holter-ECG[1]	X	X	X					
≥24h ECG-monitoring[2]	X							
12-lead-ECG	X			X				
echocardiography	X							
brain imaging	X							
vascular imaging	X							
standard laboratory testing[3]	X							
biomarkers[3]	X	X						

1 only applicable for patients in the intervention group
2 only applicable for patients in the control group
3 standard laboratory testing (e. g. cholesterol, HbA1c, Creatinine) will be performed according to local standard

🏠 clinical visit at study centre
☎ telephonic visit
ECG: Electrocardiogram
EP: endpoints
NIHSS: National Institute of Health Stroke Scale
mRS: modified Ranking Scale

1 RATIONALE

1.1 Medical background

Atrial fibrillation (AF) is the most frequent arrhythmia and may be classified as paroxysmal, persistent and permanent. The most relevant complication of AF is ischemic cardioembolic stroke. While persistent and permanent atrial fibrillation is easily diagnosed using a 12-lead ECG, the diagnosis of paroxysmal atrial fibrillation is more challenging.

Oral anticoagulation using Vitamin-K antagonists has been shown to decrease the risk of ischemic stroke by two thirds (Hart et al. 2007) and newer drugs may be even more effective (Connolly et al. 2009). However, registry data suggest that paroxysmal atrial fibrillation is largely underrepresented in anticoagulation trials and the risk of stroke and thromboembolism in paroxysmal AF is less well defined (The Task Force for the Management of Atrial Fibrillation of the European Society of Cardiology (ESC) 2010). Recent data suggest that the risk of stroke in paroxysmal atrial fibrillation is similar to persistent or permanent atrial fibrillation (Hohnloser et al. 2007, Friberg et al. 2010). Thus, detection of paroxysmal atrial fibrillation is a major challenge in primary and secondary stroke prevention. In addition, recent cost-effectiveness analyses found that prolonged (7 day) Holter ECG after cerebral ischemia saves 1 quality adjusted life year (QALY) at the price of only $ 15.000 (Kamel et al. 2010), or possibly even less (Wachter et al. 2011).

1.2 Rationale

Prolonged monitoring for atrial fibrillation (AF) in stroke patients enhances the detection rate of atrial fibrillation (Liao et al. 2007). In the Find-AF trial, we recently showed that prolonging Holter-ECG recordings from 24 hours to 7 days triples the detection rate of paroxysmal AF (Stahrenberg et al. 2010). These findings are well in line with results presented by other investigators evaluating prolonged cardiac monitoring after ischemic stroke, however, up to now we still lack clear guidelines on mode, duration, time point and selection of patients for enhanced diagnostics.

Detecting AF has a clear impact on the secondary stroke prevention strategy as anti-platelet therapy, commonly prescribed after ischemic stroke, is replaced by more effective oral anticoagulation.

Recent diagnostic trials have often focussed on selected collectives of stroke patients, especially those with cryptogenic strokes. The definition of this condition relies on stroke subclassification systems which show only moderate interrater-relyability (Chen 2011).

Furthermore, there are good reasons to justify application of extended monitoring to patients with defined stroke causes, especially atherosclerosis, which is more easily diagnosed than and shares several risk factors with paroxysmal atrial fibrillation. Thus both conditions are likely to coexist, while paroxysmal AF might often be overlooked as a result of selection bias. "Find-AF$_{RANDOMISED}$"“is the first randomised controlled trial evaluating the detection of pAF within a general stroke population.

It has long been established, that the incidence of persistent AF clearly increases with age. Data from the Find-AF trial indicates that this also applies to paroxysmal AF. In order to decrease the number needed to screen, we decided to focus extended diagnostics on elderly stroke patients >60 years of age.

As for the mode of AF-detection, multiple approaches have been evaluated (Seet et al. 2011). Unlike most other available methods, Holter monitoring offers the possibility of recording a continuous ECG, which, though requiring elaborate evaluation, should provide the highest sensitivity within a defined length of time and has been shown to be more effective than automatically triggered Loop-Recording systems (Roten et al. 2012).

Recent publications (Healey 2012, Glotzer 2009) evaluating retrospective analyses of atrial tachycardia detected by implanted devices indicate that atrial high rate episodes of short duration (>6 min.) clearly increase the risk of thromboembolism. However, it remains unclear, whether the short duration of blood stasis within the left atrial appendage during these short spells suffices to actually form thrombotic material, or whether these episodes should rather be seen as surrogates of longer AF spells. According to current guidelines atrial fibrillation has to last ≥ 30 seconds, but this cut-off is arbitrary. Within the Find-AF trial between ⅓ and ½ of the patients showed short atrial tachycardia of < 30 seconds during Holter monitoring, however the relevance of these episodes remains unclear. Some authors have actually described cardiac rhythm disorders caused by cerebral ischemia itself (Oppenheimer 2006). By further enhancing the duration and by repeating Holter monitoring we hope to provide further insight into the relevance of these supraventricular runs.

In conclusion, Find-AF$_{RANDOMISED}$ aims to focus on a cohort of patients that is clearly underrepresented in current clinical trials: An unspecific population of elderly (≥ 60 years) stroke patients.

1.3 Risk-Benefit Consideration

The detection of paroxysmal atrial fibrillation is of major relevance, as the rate of stroke and systemic embolism can be decreased by more than 50 per cent with adequate antithrombotic therapy. Holter monitoring is a medical procedure without any major physical risks, though

some may consider the procedure cumbersome. Altogether, the risk-benefit ratio for prolonged Holter monitoring shows a high benefit for those with newly diagnosed atrial fibrillation, bearing a considerably low risk of adverse events so that the study appears ethically acceptable.

2 OBJECTIVES

2.1 Primary objectives

To determine whether prolonged and enhanced monitoring increases the detection of atrial fibrillation (and atrial flutter) as compared to the usual care.

The primary endpoint is the detection of atrial fibrillation (or atrial flutter) within 6 months before occurrence of recurrent stroke or systemic embolism.

2.2 Secondary objectives

- Occurrence of Stroke (total)
- Measurement of stroke-related disability
- Stroke classification by subtype (CCS-Score)
- Percentage of guideline-adherent anticoagulation
- Occurrence of death (total), cardiovascular death, cerebrovascular death, transient ischemic attacks, myocardial infarction, systemic embolism, bleeding complications, hospitalisation for atrial fibrillation (/ flutter), stroke-related depression
- Identification of predictors of underlying occult atrial fibrillation
- Evaluation of Biomarkers (e. g. natriuretic peptides) to detect paroxysmal atrial fibrillation and recurrent stroke
- Quality of Life assessment
- Estimation of costs
- Evaluation the feasibility of prolonged monitoring procedures
- Association of detected atrial fibrillation with signs of atrial myocardial fibrosis on Cardio-MRI (Substudy at study site Göttingen only)

3 TRIAL DESIGN AND DESCRIPTION

3.1 Trial design

The trial is designed as a prospective, randomised, controlled, two-armed, parallel-group, multi-centre trial.

3.2 Requirements for participating investigators and trial sites

Participating centres must have a certified stroke unit with > 500 stroke patients per year and must apply current guidelines for stroke care.

3.3 Trial sites and number of trial subjects

Find-$AF_{RANDOMISED}$ will include a total number of 400 patients with stroke.

4 clinical trial sites will participate in this trial.

#	Name	Affiliation
1	PD Dr. Rolf Wachter	Department of Cardiology and Pneumology, University of Göttingen, Germany
2	PD Dr. Klaus Gröschel	Department of Neurology, University of Mainz, Germany
3	Prof. Dr. Pawel Kermer	Department of Neurology, Nordwestkrankenhaus Sanderbusch, Sande, Germany
4	Prof. Dr. Gerhard F. Hamann	Department of Neurology, Horst-Schmidt-Kliniken, Wiesbaden, Germany

3.4 Expected duration of trial

Duration per patient: 12 months

Expected duration of recruitment: 12 months

Expected time from first patient in to last patient out: 24 months

Expected total duration: 30 months (with 3 months preparation time and 3 months to perform follow-up course work)

3.5 Premature termination

3.5.1 Premature closure of a trial site

Premature closure of a trial site is to be considered if

- the recruitment rate is insufficient,
- if the conduct of the study is incompliant with the protocol, or
- if the quality of data is insufficient.

The premature closure of a site will be decided by the coordinating investigators and the responsible biometrician.

Investigators and trial sites that decide to abort their participation in the trial have to inform the coordinating investigator immediately. The decision should be well founded.

Details on further treatment and follow-up of patients on study have to be discussed with the coordinating investigator.

3.5.2 Premature termination of the trial

The following conditions could lead to a premature termination of the trial:

- Substantial changes in risk-benefit considerations
- New insights from other trials
- Insufficient efficacy stated in a futility analysis
- Insufficient recruitment rate.

4 SELECTION OF TRIAL SUBJECTS

4.1 Inclusion criteria

Patients must meet ALL of the following criteria:

1. Recent cerebral ischemia defined as stroke (sudden focal neurologic deficit lasting > 24h consistent with the territory of a major cerebral artery and categorised as ischemic) and/or a corresponding lesion on brain imaging.
2. Stroke symptoms started ≤ 7 days ago.
3. Age ≥ 60 years.
4. Modified Rankin scale ≤ 2 (prior to index event)

4.2 Exclusion criteria

Patients will be excluded for ANY ONE of the following reasons:

1. Known history of atrial fibrillation/flutter or atrial fibrillation/flutter on admission ECG.
2. Indication for oral anticoagulation at randomisation.
3. Absolute contra-indication against oral anticoagulation at randomisation.
4. Intracerebral bleeding in medical history.
5. Patient scheduled for Holter-ECG or cardiac Event-Recording monitoring ≥ 48 hours.
6. Significant carotid artery or vertebral artery stenosis > 50% (NASCET classification), significant intracranial artery stenosis suspicious of atherosclerotic origin or acute arterial dissection explanatory of stroke symptoms.
7. Implanted pacemaker device or cardioverter/defibrillator.
8. Life expectancy < 1 year for reasons other than stroke (e.g. metastatic cancer disease).
9. Concomitant participation in another controlled randomised trial.

4.3 Gender aspects

Patients of both genders will be included into this trial.

5 TRIAL INTERVENTIONS

5.1 Usual care (basic therapy)

Standard usual care (basic therapy) for all patients mainly targets the control of risk factors for stroke. Diagnostic procedures should be performed according to current guidelines. A standard monitoring of heart rhythm should include at least 24 hours of continuous monitoring (either monitoring on the stroke unit or 24h Holter ECG).

5.2 Enhanced and prolonged Holter monitoring

Within the intervention group, a 10 day Holter ECG will be performed during the hospitalisation period following the index event (0 months) and after 3 and 6 months thereafter. As soon as AF (or atrial flutter) has been documented, no further recording will be performed.

Some patients within the intervention arm of the trial may refuse to wear a 10-day Holter ECG a second or third time. These patients will be offered to record 2 x 30 second ECGs during the 10 day intervention period using a telemetric handheld ECG device (*Zenicor EKG-2, Zenicor Medical Systems AB, Stockholm, Sweden*). In cases when these short episodes are suspicious but insufficient to diagnose AF, the patient will be contacted to record another full ECG recording.

Intervention schedule

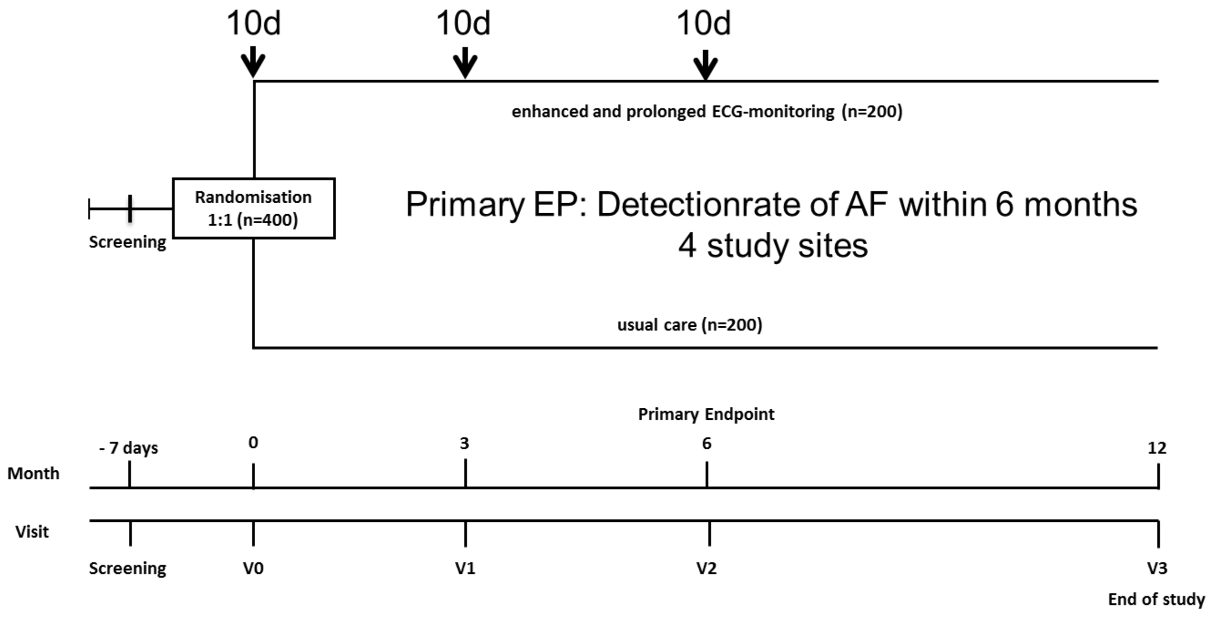

Figure 1: Intervention schedule in Find-AF$_{\text{RANDOMISED}}$. All patients in the intervention arm will receive 10 days of Holter monitoring 0, 3 and 6 months after the index event.

5.2.1 Interventional procedure(s)

Multi-Center Approach

All centres will be trained for the correct application of Holter ECG during a 1-day activation visit.

Compliance and Monitoring

Compliance will regularly be monitored and centres will be trained to increase compliance in study participants.

Contraindications or temporary interruption of intervention

In case a patient develops side effects of the Holter ECG (e.g. skin irritations due to the electrodes), appropriate measures should be applied to acquire the Holter ECG data anyway.

5.2.2 Premature termination

Currently there are no likely reasons for a premature termination of the trial. In case of unforeseen circumstances implying the study's closure, the consent of both the coordinating investigators and the biometrician is required.

5.2.3 Intervention-specific adverse events (AE/SAEs)

AE/SAEs occurring during or within 24 hours after the recording of the Holter ECG will be documented as "likely Holter ECG related". However, AE/SAEs regardless of the elapsed time between the event and the Holter ECG will be documented and evaluated.

5.3 Substudy Cardiac MRI to determine atrial fibrosis (Study Site 1 – Göttingen only)

All patients at the study site in Göttingen who are eligible for cardiac MRI will be offered to undergo this procedure. The aim of this sub study, in which we plan to include 50 patients, is to correlate atrial fibrosis with the occurrence of paroxysmal AF. Participation in this project is voluntary and patients will be allowed to be included into the Find-AF$_{\text{RANDOMISED}}$ trial even if they refuse to take part in the MRI procedure..

Details of the MRI are listed below:

Acquired scan time is approximately 15-20 min. after gadolinium injection (0.1 mmol/kg; MultiHance ®, Bracco Diagnostic Inc., NJ, USA). Contrast-enhanced 3D fast low-angle shot angiography sequence as well as a cine true-fast imaging with steady-state precession sequence are used to define the anatomy of the pulmonary veins, the left atrium (LA) and the left atrial appendage (LAA).

1.5-T Symphony or 3-T TrimTrio clinical scanner (Siemens Medical Solutions, Erlangen, Germany) with a total imaging matrix phased-array receiver coil are used in this study. The sequence for both scanners is a 3D inversion recovery (IR) fast spoiled gradient recalled sequence, that is respiratory triggered, ECG-gated and navigated, gradient-echo and fat suppressed. ECG gating is used to acquire views during the diastolic phase of the LA/LAA cardiac cycle. Time interval between the R- peak of the ECG and the start of data acquisition is defined using cine images of the LA. A transverse imaging volume and free breathing using navigator gating is used in both types of scanners.

Delayed enhancement imaging is used to detect scarring or fibrosis in left atrial wall tissue, imaging will be 5-10 minutes as well as 15-20 minutes after contrast injection.

This study protocol represents clincial routine cardiac - MRI image acquisition.

6 INDIVIDUAL TRIAL PROCEDURES

6.1 Screening

All participating trial centres will screen all consecutive outpatients and inpatients with recent ischemic stroke. Patients that appear suitable for the trial (see inclusion and exclusion criteria) should be informed about the possibility of participating. All screened patients, i.e. both those agreeing and those refusing to participate will be documented in the anonymous screening log under an assigned screening number. This document will include information on gender, age and initial NIHSS-score. The screening log is mandatory and will be supplied by the IFS Göttingen. Obviously only those willing to participate will be included into the screening phase and will undergo further diagnostic procedures after giving written informed consent (please see Appendix).

The qualifying screen visit (V0/Scr) will be structured as follows:

1) Inform the patient about the possibility of participating in the study. Hand out the patient information leaflet. Inform the patient about further diagnostic procedures to check eligibility (unless he/she refuses to participate). Inform the patient that he/she will be given time to reconsider the matter and have the opportunity to ask questions. Inform the patient that the written informed consent form must be signed before performing any of the following procedures during this visit. Obtain the patient's written informed consent on two copies of the form. Counter-sign both forms. Provide one form to the patient, store the other form in the Investigator Site File. If patients are not able to give informed consent, obtain informed consent from the legal representative (gesetzlicher Betreuer). If no legal representative has been determined yet, the patient's presumed will has to be determined by means of the SOP to be found in the appendix. Patients presumably willing to participate are included in the study and informed consent is obtained as soon as possible. If ability to give informed consent is regained or a legal representative has been determined, informed consent has to be obtained as soon as possible.
2) Confirm the patient fulfils inclusion and exclusion criteria also using the diagnostic procedures listed below.
3) Obtain a complete medical history, record concomitant medication and perform a physical examination (see Appendix for further information).
4) Obtain a 12-lead ECG.
5) Obtain an ultrasound of the carotid arteries to exclude severe stenosis.
6) Obtain information about existing indications/contraindications for oral anticoagulation.

All patients are to be listed in the anonymous screening log. Please state all reasons in detail, if a patient does not enter into the trial.

6.2 Patient information and informed consent

The investigator must explain the outline of the study to each trial subject, including its purpose, the procedures involved, the expected duration, the potential risks and benefits involved and any discomfort it may entail. Each trial subject must be informed that trial participation is voluntary, that he or she may withdraw consent at any time and that withdrawal of consent will not affect his/her subsequent medical treatment or relationship to the treating physician. The patient should be granted sufficient time to consider the participation in the study.

Informed consent should be given by means of a standard written statement (please refer to the appendix). The trial subject should read the statement and consider his/her decision before signing and dating the document. A copy of the signed document will be handed out. If written consent is not possible, oral consent can be obtained if witnessed by a signed statement from one or more persons not involved in the study, mentioning why the patient was unable to sign the form (e.g. due to arm paresis).

If no legal representative has been determined yet, the patient's presumed will has to be determined by means of the SOP to be found in the appendix. Patients presumably willing to participate are included in the study and informed consent is obtained as soon as possible. If ability to give informed consent is regained or a legal representative has been determined, informed consent has to be obtained as soon as possible.

The Informed Consent form is to be placed in the Investigator Site File. It has to be signed twice to provide another version for the participant.

The informed consent of the patient must also refer specifically to the assessment and processing of the patients' health related data. This includes the purpose and extent of the assessment and the use of his/her personal, especially the health-related data.

6.2.1 Withdrawal of informed consent

Patients may withdraw their consent to participate at any time of the trial without having to give a specific reason. Nevertheless, the reason for premature termination should be assessed after informing the patient that the question need not be answered. The patient has to be informed, that choosing not to participate or withdrawing consent will not affect his/her subsequent medical treatment or relationship to treating physicians.

In any case, the date of enrolment, as well as the date of and reason for withdrawal are to be documented.

The patient has to be informed that stored data may be deleted and samples taken may be disposed on the patients' wish.

-

6.3 Enrolment and randomisation (V0)

Eligibility of enrolment will be confirmed and the patients will be randomised at Visit 0 (week 0 of the study). Screening and V0 can be performed simultaneously, if all necessary data are available. Randomisation will be stratified by centre.

Unless recently performed, the following procedures have to take place:

1. Assessment of quality of life (SIS-16, HADS, EQ5D)
2. Assessment of health care utilisation (EQ5D- and specific health care utilisation assessment form)
3. Assessment of NIHSS and mRS by certified investigators
4. Blood sample to evaluate biomarkers and testing for Fabry's Disease
5. Echocardiography
6. 10-day Holter ECG in those randomised to prolonged Holter monitoring or 24 hours of ECG-monitoring within the control group.
7. Only applicable for study site in Göttingen: Perform Cardio-MRI in those willing to participate in this sub-investigation.

The randomisation is performed by means of sealed envelopes, which are separated in batches for each centre. The batches of envelopes are numbered consecutively with a randomisation number. The randomisation envelopes are provided by the IFS, Göttingen.

Every patient who confirms inclusion will undergo the randomisation procedure. Informed consent of patients temporarily unable to give informed consent or of patients whose legal representative remains to be determined may be obtained as soon as possible, provided that the patient himself is presumably willing to participate. If the patient is included in the study, the investigator will open the next available envelope in the appropriate batch, which contains a randomisation file. This file provides the group allocation and has to be dated and signed by the investigator. The signed randomisation file has to be stored in the Investigator Site File

(ISF).

6.3.1 Violation of eligibility criteria

If a patient has been included into the study and it turns out that he/she violated the eligibility criteria, proceed as follows:

In case the patient has not been randomised (as is the usual case), cancel the participation in the trial entirely. Since a patient ID code has already been assigned, notify the withdrawal in the patient list (CP) and do not use the applied ID code for any other patient.

If the patient has already been randomised, contact the coordinating investigator/sponsor and IFS data management immediately. In general, the violation of eligibility criteria will not lead to premature withdrawal of the patient from the study. Cancel the participation immediately only in cases when the violated criteria represent an additional risk for the patient, or if the patient has been randomised by error, i.e. without his/her consent. In all other cases, proceed per protocol and await instructions from the coordinating investigator/ sponsor.

6.3.2 Clinical Course

Specific information on the patient's clinical course during primary hospitalisation should be documented. This includes information on the patient's survival status, clinical events and procedures (lysis therapy, endovascular thromboectomy, pneumonia, and antibiotic treatment), documentation of atrial fibrillation/flutter and guideline adherence regarding antiplatelet/anticoagulation therapy. The data should be obtained and entered into the eCRF shortly after the patient is discharged from hospital.

6.4 Trial visits

6.4.1 Baseline visit (Scr/V0)

The procedures to be performed at the baseline visit (Scr/V0) are explained in detail in chapter 6.3 (enrolment and randomisation). Please refer to this chapter.

6.4.2 Follow-up visits 1, 2 and 3 (V1, V2 & V3)

During follow-ups 1 and 2 the following procedures will be performed:

1) Obtain current medical status including possible hospitalisations (if possible, acquire medical report) and concomitant medication, measure the vital signs. Assess guideline

adherence. Document any adverse events (in case of SAEs check whether patient was on any Boehringer-Ingelheim product at the time of the event, if that is the case proceed as described in the AE/SAE section).

2) Obtain NIHSS and MRS, assessed by certified investigator.

3) Only applicable for V1 and V2: Perform 10-day Holter ECG on patients in the enhanced and prolonged Holter monitoring arm of the study. If atrial fibrillation (or atrial flutter) has been detected by Holter ECG or by 12-channel ECG and is confirmed by the central independent endpoint adjudication committee, the intensified monitoring will be terminated. In cases when patients predetermined to receive Holter monitoring refuse to wear the device again, they will be offered to record repetitive ECGs using a hand-held single channel ECG-device twice a day (Zenicor EKG-2, Zenicor Medical Systems AB, Stockholm, Sweden) for 10 days.

4) Only applicable for V1: Take a blood sample for assessment of biomarkers and assess quality of life (both as described in detail in 6.3).

5) Only applicable for V2 and V3: Assess health care utilisation (as described in detail in 6.3).

6) Only applicable for V3: Perform 12-lead-ECG.

6.4.3 Extended 3-year follow-up (V4 – V7)

After the final clinical visit at 12 months, each patient will be continually followed-up for another two years, i.e. until three years after index event. These additional follow-up visits (V4-V7) will be telephonic and will take place every 6 months. The following data will be acquired:

1) Obtain current medical status including possible hospitalisations (if possible, acquire medical report) and concomitant medication. Assess guideline adherence. Document any adverse events (in case of SAEs check whether patient was on any Boehringer-Ingelheim product at the time of the event, if that is the case proceed as described in the AE/SAE section).

2) Obtain mRS.

3) Document, whether diagnosis of atrial fibrillation/flutter has been made. If it has, try to acquire the ECG-document, on which the diagnosis was founded.

4) Assess healthcare utilisation (as described in detail in 6.3).

6.5 Processing and storage of blood samples, evaluation of Biomarkers and testing for Fabry's disease

An addition 8 vacutainers containing a maximum of 56,4 ml of blood will be collected from each patient: 2 x 9 ml EDTA, 2x 2,7 ml EDTA, 2 x 7,5 ml serum and 2 x 9 ml lithium-heparin. All samples except one large and the two small EDTA-samples will be centrifuged and the supernatant will be filled into aliquots immediately, which will then be stored at -20°C or lower.

These samples will be used for no purpose other than explorative study-related research, though the evaluation of blood samples is not part of the eCRF.

After the trial's completion they will mainly serve to analyse biomarkers with the specific goal of identifying predictors of atrial fibrillation and defined outcome parameters in stroke patients. Evaluated markers will include a series of known markers of cardiovascular impairment, such as natriuretic peptides (e.g. NT-pro-BNP, NT-pro-ANP), markers of myocardial damage (troponins, heart-type fatty acid binding protein), and growth-differentiation factor (GDF-15). New approaches, such as microRNA structures (and thus including analysis of genetic material), which have recently been associated with atrial fibrillation, may also be evaluated.

The remaining blood samples will be kept and stored for possible future evaluation of new specific approaches within this field of research. All samples will be stored according to current legal regulations.

To our best knowledge, none of the evaluated markers will impart therapeutic consequences or information of major relevance to the patient (incidental identification of chronic diseases, genetic disorders, etc.).

Blood samples obtained for Core Lab analysis in Göttingen will be stored at -80°C, the other centres will send the samples to the core lab in Göttingen at specific time points. Specifically prepared containers for mailing the samples and a handling guideline will be provided to each trial centre by the study coordinator.

All participators will be offered the option of screening for Fabry's disease, a rare genetic cause of cerebral ischemia. However, the participation in this subanalysis is not mandatory and those refusing to undergo genetic testing will nevertheless be allowed to take part in the Find-$AF_{RANDOMISED}$ trial. Each patient undergoing this test (or their legal representative) will be asked in advance, whether or not he/she wishes to be informed about a positive result. Refusal to be informed will be documented on the patient information form. For this analysis, the small (2,7 ml) vacutainers containing EDTA-blood will be immediately forwarded to the "Albrecht-Kossel-Institut" in Rostock, Germany, in unprocessed form. All results will first be forwarded to the study coordination centre in Göttingen. From there, the positive results will be

handed on to each trial centre. If the patient in question (or his/her legal representative) wishes to be informed about his/her result, the local investigator will arrange an appointment at a local genetic counselling centre, where the diagnosis will be delivered and all necessary information will be provided. On request of the patient/legal representative, the results will also be forwarded to the treating physician, as therapeutic options will not be part of this trial.

6.6 Premature termination of trial participation

Each premature termination of the trial procedures as well as every premature termination of follow-up has to be documented by the responsible investigator. If possible, the date and circumstances of as well as the reasons for termination should be documented in detail and passed on to the IFS Göttingen.

7 ADVERSE EVENTS AND SERIOUS ADVERSE EVENTS (AE/SAE)

7.1 Definitions

7.1.1 Adverse Events

An Adverse Event (AE) is any untoward medical occurrence in a patient or clinical investigation subject administering an interventional procedure. It does not necessarily have to have a causal relationship with this treatment. An AE can therefore be any unfavourable and unintended sign (including abnormal laboratory findings), symptom, or disease temporally associated with the administration of the interventional procedure, whether or not it is considered to be related to the interventional procedure. (ICH-Guideline E2A)

Adverse events encompass illness, signs of illness (including pathological laboratory findings) and symptoms that initiate during the trial or previous conditions that become worse.

7.1.2 Serious Adverse Events (SAE)

A Serious Adverse Event (SAE) is any occurring medical condition that

- results in death,
- is life-threatening,

  NOTE: The term "life-threatening" in the definition of "serious" refers to events in which the patient was actually at risk of death at that; it does not refer to an event, which hypothetically might have been life threatening in a more severe case.
- requires in-patient hospitalisation or prolongation of existing hospitalisation or
- results in persistent or significant disability/incapacity.

7.1.3 Relevant Serious Adverse Events in the context of the Find-AF$_{\text{RANDOMISED}}$-Trial

Relevant Serious Adverse Events in the context of the Find-AF$_{\text{RANDOMISED}}$-Trial are those events fulfilling the criteria of SAEs as quoted above, that could be related to the performed study-specific procedures. Therefore all SAEs occurring after application of the Holter-Monitor and until 2 hours after the Holter device is removed are considered relevant.

7.2 Documentation and Reporting

7.2.1 General Documentation and Reporting

All occurring SAEs must and AEs should be documented in the specific AE/SAE-form that is part of the eCRF. In the context of the present study, relevant adverse events are those, which could arise from the prescribed Holter monitoring in the intervention arm, as defined above. In addition to being documented within the eCRF, all those events meeting the criteria of relevant SAEs (7.1.3) have to be documented on the manual SAE-form (which can be downloaded within the eCRF data base). The completed form has to be submitted to the sponsor (IFS) by fax (fax number 0049-551-39171293) within 24 hours after gaining knowledge of the event.

In case of an SAE, the patient's medication has to be screened for any BOEHRINGER INGELHEIM product (see 7.2.2).

7.2.2 Reporting to BOEHRINGER INGELHEIM:

The Investigator shall report all: SAEs and non-serious AEs occurring at the same time and/or which are medically relevant for a reported SAE occurring after application of any BI DRUG taken in this trial within 24 hours by fax or other secure method to the BI Unique Entry Point.

The Coordinating Investigator shall inform BI about all AEs occurring with any BI Drug by means of the final Study Report.

8 BIOMETRICAL ASPECTS

8.1 Randomisation algorithm

Patients will be randomised 1:1 for prolonged Holter monitoring or the control group (usual care). Randomisation will be stratified by each centre. Lists of separate randomisation sequences for each stratum will be generated using SAS® 9.3 with the random functions ranperm and rantbl. Permuted block-randomisation with variable block-length is applied to generate the randomisations lists, which are the base of envelope serial print.

Initially, each centre receives 100 randomisation envelopes. Further envelopes can be delivered in addition if required.

8.2 Endpoints

8.2.1 Primary endpoint

The primary endpoint is the detection of atrial fibrillation (and atrial flutter) within 6 months before occurrence of recurrent stroke or systemic embolism. Atrial fibrillation is defined as any arrhythmia fulfilling the criteria for atrial fibrillation (the same applies to atrial flutter) as given in the current guidelines (including a minimum duration of at least 30 seconds). The blinded endpoint adjudication committee will adjudicate all episodes potentially qualifying for the primary event.

Detection of atrial fibrillation or flutter is defined as a binary variable (yes or no) regardless of the time of detection, the duration of monitoring according to the study protocol and the source of information in case of report of atrial fibrillation or flutter. In particular, atrial fibrillation or flutter proven by occasional ECG outside the study protocol but within the time frame of six month will imply the primary endpoint to be "yes". On the other hand, if Holter monitoring according to the study protocol is discontinued and, therefore, detection of atrial fibrillation or flutter that might have occurred is impossible, the primary endpoint is set to "no".

The reasoning for this definition is that prolonged Holter monitoring is being offered to the patients as an opportunity that can be accepted or not. We aim to investigate how many events of atrial fibrillation or flutter can be detected by this approach on top of usual care.

Thus, we are aiming to evaluate the performed procedures within a real world setting, rather than estimating theoretic rates of events that could be detected, if all subjects fully comply with the offered monitoring procedures.

8.2.2 Secondary endpoints

Major secondary endpoints

1. Detection of atrial fibrillation (/flutter) as defined for the primary endpoint after 12 months.
2. Detection of atrial fibrillation (/flutter) as defined for the primary endpoint with the exception that hospitalisation for atrial fibrillation (/flutter) will be considered as censoring.
3. Recurrent stroke or systemic embolism after 12 months.
4. Total death after 12 months.

Other secondary endpoints (all after 12 months)

1. Cardiovascular death
2. Cerebrovascular death
3. Transient ischemic attack
4. Myocardial infarction
5. Bleeding complications
6. Quality of Life
7. Incremental detection of atrial fibrillation or flutter in the extended Holter monitoring periods after 3 and 6 months
8. Costs
9. Feasibility of monitoring procedures
10. Association of detected atrial fibrillation with signs of atrial myocardial fibrosis in Cardio-MRI (study site in Göttingen only)

Definitions of endpoints:

New onset of atrial fibrillation will be defined as any period of 'absolutely' irregular RR intervals without distinct P waves and (if visible) with a variable atrial cycle length usually <200 ms. Any episode that lasts sufficiently long for a 12-lead ECG to be recorded or at least 30s on a rhythm strip will be considered atrial fibrillation.

New onset of atrial flutter will be defined as any episode of supraventricular tachycardia defined as such by an expert in electrophysiology and lasting sufficiently long for a 12-lead ECG to be recorded or > 30s on a rhythm strip. These episodes typically show an atrial frequency of 250-340 beats/minute. Typical atrial flutter shows a sawtooth pattern, best visualised in leads II, III, aVF or V_1.

Deaths will be subdivided into vascular (e.g., stroke, embolism, or acute MI) and nonvascular (e.g. malignancy or haemorrhage) causes.

Guideline-adherence. Patients should be treated according to current guidelines.

This includes anticoagulation, if AF is detected during the study period. All efforts (informing the patient, the caregivers and all treating physicians) should be made to maintain an optimal INR-range if applicable. In cases when patients do not receive guideline-recommended therapy during the study

period, the study personnel will provide detailed information. The major points of the current treatment guidelines will be checked by the study personnel during each visit and documented within the eCRF.

Myocardial infarction (MI) will be defined as in the RE-LY trial (Ezekowitz et al. 2009). In summary, the definition asks for 2 of the 3 following criteria in patients without percutaneous coronary intervention (PI) or coronary artery bypass (CABG): typical severe chest pain or related symptoms/signs (e.g. ST changes of t-wave-inversion on ECG suggestive of MI), elevation of troponin or creatinin-kinase-MB or development of significant Q-waves in at least 2 adjacent ECG-leads. After PCI or CABG: elevation of troponin or CK-MB or development of significant Q-waves. Silent myocardial infarction: retrospective development of significant Q-waves.

The occurrence of bleeding under antithrombotic therapy will be subdivided into minor and major. Major bleeding is defined as a reduction of haemoglobin concentration by at least 20 g/L, transfusion of at least two units of blood or symptomatic bleeding in a crucial area or organ. All other bleedings will be regarded as minor.

The quality of life will be measured according to the scheduled visits using the Stroke Impact Scale (SIS-16). The primary endpoint for quality of life will be the disease-specific physical quality of life as measured by the SIS-16. In addition, the HADS will be used to check for stroke-associated depression and anxiety that typically affect quality of life outcomes.

Costs: Rational resource allocation to match needs and investing in the most efficient diagnostic and therapeutic strategies is becoming increasingly important. Costs are categorised into direct, indirect, and intangible costs. Direct costs include all medical resources that are directly involved in the particular intervention, including the costs for pharmaceuticals, hospitalisations, rehabilitation, outpatient contacts etc. Indirect costs reflect the loss to society as a result of lost or impaired ability to work due to morbidity or death. Resource use will be assessed by using a standardised questionnaire.

The cost-effectiveness will be measured using the incremental cost-effectiveness ratio (ICER). The ICER is the difference between the costs of two technologies, divided by the difference of their health outcomes. A common problem associated with cost-effectiveness analyses is the comparability of interventions given qualitatively different health risks and improvements. Using a common metric of health effects and utilities such as quality-adjusted life years (QALY) improves the comparability of ICERs across different interventions and diseases (cost-utility-analysis). The EQ-5D is an index instrument used in quality of life surveys that helps generate utilities to perform such cost-utility analyses.

Feasibility of Monitoring Procedures: The assessment of feasibility will be based on a specific questionnaire, which will allow documentation of specific adverse effects, general confinement, duration of recording and reasons for aborting the procedures.

Atrial Myocardial Fibrosis: Atrial myocardial fibrosis and atrial size will be determined by means of a specific cardiac-MRI protocol (only in a selected amount of patients at the study site in Göttingen).

Remark: Total mortality will not be included as primary endpoint seeing there is no proven difference in mortality between patients receiving oral anticoagulation or compared to aspirin (De Catarina et al, 2010).

8.3 Statistical hypothesis

The detection of atrial fibrillation (/ flutter) within six months (Kaplan-Meier-Estimate) is higher in the prolonged and enhanced monitoring arm as compared to the control arm.

8.4 Sample Size Discussion

8.4.1 Consideration of effect size

In the Find-AF trial (Stahrenberg et al. 2010), prolonged monitoring of up to 7 days resulted in a AF detection rate of 12.5 % of the patients initially presenting in sinus rhythm, whereas the detection rate after 24 hours was only 4 %. In this study, we will further intensify the ECG-monitoring (10 day Holter at months 0 and 3 and 6) and thus expect to find AF in at least 15 % of the patients.

We decided to include 400 patients into this trial. We assume a dropout rate of 15 %, leaving 340 patients. This sample size would give our study a power of 83 % to detect a difference between 15 % detection rate (intensified monitoring) and 5 % (usual care) and 98 % power to detect a difference between 20 % (intensified monitoring) and 5 % (usual care).

Compared to published data (Seet et al. 2011, Liao et al. 2007), our estimates are rather conservative and the detection rate using intensified monitoring might well be higher (Wallmann et al. 2007).

8.4.2 Level of significance and power

One sided type I error of 0.025.Power 0.83 for a difference of 15 % versus 5 % and 0.98 for a difference of 20 % prevalence versus 5 %.

8.4.3 Drop-outs

We estimate a censoring rate of 15% (withdrawal of consent, death, recurrent stroke).

8.4.4 Sample Size Calculation

To meet the assumptions above, we need to recruit n=400 patients (200 per group), i.e. 340 patients without the dropouts.

8.4.5 Power Analysis

A power of 0.98 (see above) was chosen in order to achieve satisfactory power also for the secondary endpoint of the discovery of atrial fibrillation (and atrial flutter) with censoring of hospitalisation for atrial fibrillation (or atrial flutter).

8.5 Statistical methods

8.5.1 Analysis population

Primary analysis: all patients randomised, to be analysed in the arm they were randomised for.

Secondary analyses: all patients with complete data needed for the respective analyses.

8.5.2 Planned analysis methods

The primary analysis is carried out by the long-rank test for the Kaplan-Meier estimates for the time to first documentation of atrial fibrillation (and atrial flutter). Death, recurrent stroke and systemic embolism will be considered censoring. The predictor variable is the group variable. In addition, multiple binary logistic regression for documentation of atrial fibrillation (and atrial flutter) within 6 months is carried out including age, severity of the event qualifying for inclusion, baseline CHADS-2 score, the randomised group and the time of observation as predictor variables. All analyses will follow the intention-to-treat principle.

Secondary analyses will include: Time to first documentation of atrial fibrillation (/flutter) as defined for the primary endpoint after 12 months, time to first documentation of atrial fibrillation (/flutter) as defined for the primary endpoint with the exception that hospitalisation for atrial fibrillation (/flutter) will be considered as censoring, recurrent stroke or systemic embolism after 12 months, total death after 12 months, cardiovascular death, cerebrovascular death, transient ischemic attack, disabling stroke, stroke by subtype, myocardial infarction, bleeding complications, quality of life and costs.

8.6 Interim Analysis

There will be no interim analysis to evaluate preliminary termination of the trial early.

8.7 Final Analysis

The final analysis will be carried out when all recruited patients have had their 12-months follow-up examination or have dropped out of follow-up.

9 ETHICAL, LEGAL AND ADMINISTRATIVE ASPECTS

All persons participating in the conduct of the trial commit themselves to observe the Declaration of Helsinki (Version Sommerset West 1996), as well as all pertinent national laws and the ICH guidelines for Good Clinical Practice (GCP) issued in June 1996 and CPMP/ICH/135/95 from September 1997 (as far as applicable to this non-pharmacological trial).

9.1 Submission

Prior to beginning biomedical research projects on human beings, all physicians are to be audited on ethical and legal questions by an ethics committee (Musterberufsordnung für die deutschen Ärztinnen und Ärzte (MBO-Ä) §15) at a „Landesärztekammer" or a Medical Faculty.

The trial will be submitted for approval to the ethics committee responsible for the coordinating investigator. After the first ethics committee approves, the study documents will be handed on to all ethics committees responsible for the participating sites for local approval. The individual study sites may begin recruiting patients only after their local ethics committee has approved.

9.2 Protocol Amendments

Changes requiring further approval and positive review by the ethics committee include:

- changes that could affect patient safety, such as essential changes in therapeutic or diagnostic procedures,
- changes concerning risk benefit considerations,
- additional data collection or statistical evaluations that necessitate changes in the informed consent form,
- new scientific data leading to changes within the rationale or the expected significance of the trial and
- significant changes concerning leadership or conduct of the trial.

Amendments should be made only in exceptional cases given mutual agreement among the coordinating investigators and the biometrician. Any amendment must be set out in written form, stating the essential reasons and has to be signed by all parties involved. The amendment then becomes part of the study protocol, and will be filed in the Trial Master File (TMF). Amendments that could have an impact on the well-being of the subject (major amendments), such as the use of additional invasive diagnostic procedures, require an additional approval by the Ethics Committee (EC). Furthermore, all trial subjects possibly affected by the amendment in question will have to sign an additional informed consent form. Minor changes will only be submitted to the Ethics Committee in written form.

10 DATA HANDLING AND RECORD KEEPING

10.1 Case Report Forms (CRF)

The clinical eCRF will be provided by the “Institut für anwendungsorientierte Forschung und klinische Studien (IFS), Göttingen” in electronic form. Investigators will connect to the database via Internet and insert the data directly. Each eCRF page should be completed as soon as possible after the respective visit and then be signed electronically. The signatures serve to verify the information contained in the eCRF and to rule out falsification.

A paper version of all eCRF sheets will be available for download within the eCRF database. This paper CRF may serve as patient source data, which will be overseen by the trial monitor.

It is the investigator’s responsibility to ensure that the trial is executed in accordance with the principles of GCP and the study protocol, and that data are entered into the database for this study in a correct and complete way. Data are entered directly into the eCRF, as soon as possible.

All patient related data will be recorded under a pseudonym. The Investigator will compile a confidential list, which relates these patient numbers to the patient’s full name. This list will only be accessible to the study team and the monitor. Original patient files may be viewed by monitors, auditors and inspectors.

The local principal investigator carries the final responsibility for the accuracy and authenticity of all clinical and laboratory data entered in the eCRF at all times.

Investigator Site File

All essential documents will be kept in the Investigator Site File (ISF) which will be stored at the Trial Site in accordance with the principles of GCP.

Data sources:

- Clinical or demographic data → patient file.
- Echo data → video/digital recordings and printouts.
- ECG and Holter ECG data → print-out.

10.2 Data Management

The study database is generated with secuTrial®, which is compliant with the necessary regulations. Data capture is web-based and will be performed at each site. The transmission of data is protected by SSL encryption. Electronic data will be saved and identified with the patients' pseudonym.

Every person, who is authorized for data capture or data monitoring will get an own user account with password. The IFS keeps a listing of all users which must be updated, if needed. Every user of the database is required to keep his account confidential without forwarding it to others. Automatic checks of data range, validity and consistency are implemented within the database.

Possible queries will only be allowed to be answered by authorized personnel. After the last patient is out and data capture and corrections are finished, the database will be locked. This procedure will be recorded in written form. If a patient withdraws the consent to participate and declares that stored data have to be deleted, this is done immediately.

The "Institut für anwendungsorientierte Forschung und Klinische Studien" (IFS), Göttingen, will be responsible for this part.

10.3 Archival storage

The investigators have to arrange the retention of the subjects' identification codes for 10 years (unless legal regulations require longer storage) after completion or termination of the trial. Patient files and other source data will be kept for the maximum period of time permitted by the hospital.

The coordinating investigator or others dealing with the data shall retain all other documentation pertaining to the trial for 10 years (unless legal regulations require longer storage). These procedures include:

- the protocol including the rationale, objectives, statistical design and methodology of the trial, as well as the conditions under which it is performed and managed and details of the investigational procedures used,
- standard operating procedures,
- all written opinions on the protocol and procedures,
- the final report,
- the case report forms,
- the audit certificate(s), if available,
- all other relevant documents of the trial master file, according to the ICH-GCP guideline.

Any changes of data ownership have to be documented. All data have to be made available if requested by relevant authorities.

11 REFERENCE EVALUATIONS

Central Holter ECG core lab:

Dr. Joachim Seegers, Göttingen, will head the centre core lab for Holter ECG analysis. It will be aimed to provide Holter ECG analyses within two working days upon receipt of the memory cards. The primary analysis will be carried out by study personal specially trained in the evaluation of Holter-ECGs with a specific focus on detecting atrial fibrillation (or atrial flutter). After primary detection of atrial fibrillation (or atrial flutter), the recording in question will be validated by an experienced physician (Prof. Zabel – Göttingen, Prof. Laufs – Homburg/Saar, Prof. Conen – Basel).

Collection of ECG recordings:

All new cases of atrial fibrillation (and atrial flutter) should be documented on any type of ECG recording (Holter-ECG, monitor ECG, 12 lead-ECG or other) in order to allow external validation. Therefore a copy of all ECG printouts on which a new diagnosis of atrial fibrillation (-flutter) is based will be collected centrally. In cases when atrial fibrillation (/flutter) is diagnosed outside study procedures, all efforts should be made to obtain the recording to validate the diagnosis. As soon as the recording is obtained by the investigator, a copy will be forwarded to the central ECG collection, before storing the original in the Investigator site file. Before handing on any ECG material, however, the investigator has to ensure, that the patient's file number (and if possible the date of the recording) is shown on the document, while no personal information (especially name and date of birth) may remain identifiable.

Final validation of AF diagnosis by the external endpoint adjudication committee

Three external experts in Electrophysiology (Prof. Zabel – Göttingen, Prof. Laufs – Homburg/Saar, Prof. Conen – Basel) will validate the collected episodes of atrial fibrillation (and atrial flutter).

12 SUPERVISION OF CLINICAL TRIAL

12.1 Direct access to source data

According to ICH-GCP and the applicable German laws, the principal investigator has to authorise third parties to access the trial site and grant insight into the medical records of the trial subjects (source data). Authorised parties include the clinical trial monitors, auditors and other authorised employees of the sponsor, as well as members of the local or federal authorities. All these persons are sworn to secrecy.

12.2 Monitoring

The investigators permit the monitor access to any or all of the study materials needed for source data verification and proper review of study progress in order to ensure adequate data collection and adherence to the protocol.

Furthermore, the investigators agree to cooperate with the monitor and to provide all required information, whenever necessary. This includes access to all records connected to the study, such as relevant original patient data. The responsibilities of the investigators include the keeping of patient health records as complete as possible, including e.g. the documentation of patient history, concomitant diseases, enrolment into the study, study visit data, results from investigations and AEs. Within pre-defined intervals, the monitor will further be allowed to carry out data verification and the comparison of data with relevant original patient data in accordance with the standard operating procedures (SOPs) of the Institut für anwendungsorientierte Forschung und klinische Studien GmbH (IFS), Göttingen, and ICH-GCP guidelines, in order to ensure adherence to the protocol and continuous recording of data. All original diagnostic findings, which act as source data for the eCRF and the database, respectively, will be examined. By signing the ICD, the participating patient/legal guardian agrees with the examination measures as stated above.

During the clinical trial, monitoring will be carried out by an external monitor of the IFS and will follow the IFS' SOPs and the respective ICH-GCP guidelines. After specific risk assessment, an adapted monitoring concept will be established. Monitoring includes site initiation, regular monitoring visits after enrolment of the first patient, as well as a close out visit after completion of the last study-specific assessment. During the regular monitoring visits, a 100% benchmark data check will be performed for all patients. In addition, a 100% data comparison with the remaining original data will be performed for at least 5% of patients.

The monitor sees that the protocol is followed. In cases of conspicuous findings, such as violations of documentation rules or protocol violations, countermeasures will be initiated after prior consultation with the Principal Investigator and the Sponsor (or its representative).

The responsible monitor is required to keep all information confidential and to ensure that the rights of the involved persons to integrity and protection of privacy are guaranteed.

12.3 Audits

In order to guarantee that the conduct of the study is in accordance with ICH-GCP and the national laws, the sponsor reserves the right to audit selected trial sites. The auditor will be independent from the staff involved in the proceedings of this clinical study.

The investigator agrees to give the auditor access to all relevant documents for review.

13 DATA PROTECTION

Within this study, personal data of the trial subjects (name, initials, date of birth, address) and data regarding the therapy as well as the course of disease will be collected.

The trial subject will be informed that all data will be stored electronically and handled with strict confidentiality. Throughout the process of documentation and evaluation the subjects will be referred to by their individual patient number only, while the subjects' names will be kept secret by the investigator. The subjects will be informed about the temporary storage of their address and phone number and must agree by signing the appropriate consent form.

The investigators are obliged to keep all study data and information confidential and to use those data only in context with the persons involved in the trial conduct. Study material or information collected in this trial must not be available to third parties, except for official representatives of the sponsor or regulatory authorities.

Data will be processed in the "Institut für anwendungsorientierte Forschung und Klinische Studien" (IFS), Göttingen in cooperation with the department of medical informatics (Abteilung für Medizinische Informatik, Universität Göttingen), according to the written safety concept of these institutions. Access to the data will be strictly limited to authorised persons. Loss of data is excluded due to extensive back-up procedures. All legal requirements concerning data protection and confidentiality will be respected. All authorised persons are sworn to secrecy.

In case consent is withdrawn and the patient declares that stored data have to be deleted, this is done immediately.

Collected personal data will be stored anonymously for 10 years (unless legal regulations require longer storage) after completing the study's aim/after finishing all concomitant scientific projects, unless there are other regulatory or contractually archiving periods.

Declaration to data protection

During data entry, handling and analysis in the "Institut für anwendungsorientierte Forschung und klinische Studien" (IFS), Göttingen, all requirements of the data protection act will be taken into account. Access to the data is strictly limited to authorised persons. Data are protected against unauthorised access.

14 ADMINISTRATIVE AGREEMENTS

14.1 Adherence to the protocol

The clinical trial will be conducted in accordance with local laws and ICH guidelines for Good Clinical Practice (GCP) issued in June 1996 and CPMP/ICH/135/95 from September 1997, taking into account the Declaration of Helsinki and all its revisions.

Protocol violations are any deviations from the procedures outlined in this document:

- missed evaluations/ incorrect timing of evaluations
- non-compliance with study intervention.

After a patient has been enrolled, it is the investigator's responsibility to make a reasonable effort to correct any protocol violation in order to keep the subject in the study.

Major protocol violations during the course of the study will be reported to the coordinating investigator immediately. The nature of these violations will be defined in the monitoring manual. All protocol violations will be listed and discussed with the coordinating investigator and biometrician prior to statistical analysis.

The investigator will make every effort to record the data according to the protocol. Under practical working conditions, however, some minor variations may occur due to circumstances beyond the control of the investigator. All such deviations will be documented in the records, together with the reason for their occurrence, and, if appropriate, detailed in the study report.

14.2 Funding and Insurance

The trial is funded by Boehringer Ingelheim. This grant is unrestricted and the company will take no influence on the protocol, the conduct of the study, the evaluation of data and the publication of the study results. Insurance for trial subjects is not necessary as the intervention is only observational and the measurements are harmless and non-invasive.

14.3 Publication policy

Publication policy will follow the official publication rules of the DFG. This includes that results that have been obtained with funding for Find-$AF_{RANDOMISED}$ have to be published in any case and as soon as possible. In general, participants who contributed to Find-$AF_{RANDOMISED}$ will be eligible for co-authorship. Co-authorship will be related to the input of the investigator of a specific project. Persons that are substantially involved in the study, such as laboratory staff, study nurses etc. will be eligible for naming in the acknowledgment section. A more detailed

regulation of publication policy will be defined by the coordinating investigators. In order to publish the study in a high-ranked journal, it will be registered at http://controlled–trials.com after approval by the ethics committee and prior to starting the enrolment.

15 REFERENCES GENERAL

Cancer Therapy Evaluation Program, Common Terminology Criteria for Adverse Events, Version 3.0, DCTD, NCI, NIH, DHHS; March 31, 2003 (http://ctep.cancer.gov), Publish Date: December 12, 2003

Committee for proprietary medicinal products (CPMP). Points to consider on multiplicity issues in clinical trials. EMEA, London 19 September 2002 CPMP/EWP/908/99.

Declaration of Helsinki: Guiding Physicians in Biomedical Research Involving Human Subjects. Adopted by the 18th World Medical Assembly, Helsinki (Finland), June 1964. Last amendment by the 48th General Assembly, Somerset West (Rep. of South Africa) 1996

European Commission (2004.04): Detailed guidance on the collection, verification and presentation of adverse reaction reports arising from clinical trials on medicinal products for human use, revision 2

Gesetz über Verkehr mit Arzneimitteln (Arzneimittelgesetz – AMG) in der Fassung der Bekanntmachung vom 05. August 2004 (BGBl. I S. 2031)

International Conference on Harmonisation of Technical Requirements for the Registration of Pharmaceutical Products for Human Use: ICH Harmonized Tripartite Guideline, "Guideline for Good Clinical Practice". Recommended for Adoption at Step 4 of the ICH Process on 1 May 1996. www.ifpma.org/ich5e.html#GCP

International Conference on Harmonisation of Technical Requirements for the Registration of Pharmaceutical Products for Human Use: ICH Harmonized Tripartite Guideline, "Clinical Data Safety Management: Definitions and Standards for Expedited Reporting". Recommended for Adoption at Step 4 of the ICH Process on 27 May 1994. www.ifpma.org/ich5e.html#Safety

Verordnung über die Anwendung der Guten Klinischen Praxis bei der Durchführung von klinischen Prüfungen mit Arzneimitteln zur Anwendung am Menschen (GCP-Verordnung – GCP-V) in der Fassung der Bekanntmachung vom 12.August 2004 (BGBl. I S. 2081)

16 CONFIRMATION OF THE FINAL PROTOCOL

The signatories declare that they agree to conduct their responsibilities within this study in accordance with local law, the declaration of Helsinki, ICH-GCP and the study protocol as presented.

Coordinating investigator	____________________ Date	____________________ Signature
Coordinating investigator	____________________ Date	____________________ Signature
Sponsor	____________________ Date	____________________ Signature
Biometrician	____________________ Date	____________________ Signature

17 PROTOCOL AGREEMENT

(To be signed by the principal investigator before/ during site initiation and prior to recruitment of patients respectively)

Herewith I declare that I have read and understood the protocol and agree to conduct the study accordingly. I will ensure that all persons assisting with the study under my supervision are adequately informed about the protocol, the investigational product and their duties.

Date: ___________________________

Signature of principal investigator: ___________________________

Affiliation /address (stamp): ___________________________

____

____

____

17.1 References related to Find-AF$_{RANDOMISED}$

Chen PH, Gao S, Wang YJ, Xu AD, Li YS, Wang D. Classifying Ischemic Stroke, from TOAST to CISS. CNS Neurosci Ther. 2012 Jun;18(6):452-6.

Connolly SJ, Ezekowitz MD, Yusuf S, Eikelboom J, Oldgren J, Parekh A, Pogue J, Reilly PA, Themeles E, Varrone J, Wang S, Alings M, Xavier D, Zhu J, Diaz R, Lewis BS, Darius H, Diener HC, Joyner CD, Wallentin L; RE-LY Steering Committee and Investigators. Dabigatran versus warfarin in patients with atrial fibrillation. N Engl J Med 2009;361:1139-1151.

De Caterina R, Connolly SJ, Pogue J, Chrolavicius S, Budaj A, Morais J, Rendal G, Yusuf S on behalf ot the ACTIVE Investigators.

Mortality predictors and effects of antithrombotic therapies in atrial fibrillation: insights from ACTIVE-W. Eur Heart J 2010;31:2133-2140.

Ezekowitz MD, Connolly S, Parekh A, Reilly PA, Varrone J, Wang S, Oldgren J, Themeles E, Wallentin L, Yusut S. Rationale and design of RE-LY: Randomized evaluation of long-term anticoagulant therapy, warfarin, compared with dabigatran. Am Heart J 2009;157:805-810.

Feng W, Hendry RM, Adams RJ. Risk of recurrent stroke, myocardial infarction, or death in hospitalized stroke patients. Neurology 2010;74:588-593.

Fine JP, Gray RJ. A proportional hazards model for the subdistribution of a competing risk. JASA 199;94:496-509.

Friberg L, Hammar N, Rosenqvist M. Stroke in paroxysmal atrial fibrillation: report from the Stockholm Cohort of Atrial fibrillation.S troke 2010;31:867-875.

Gillis AM, Rose MS. Temporal patterns of paroxysmal atrial fibrillation following DDDR pacemaker implantation. Am J Cardiol 2000;85:1445-1450.

Glotzer TV, Daoud EG, Wyse DG, Singer DE, Ezekowitz MD, Hilker C, Miller C, Qi D, Ziegler PD. The relationship between daily atrial tachyarrhythmia burden from implantable device diagnostics and stroke risk: the TRENDS study.. Circ Arrhythm Electrophysiol. 2009 Oct;2(5):474-80.

Hart RG, Pearce LA, Aguilar MI. Adjusted-dose warfarin versus aspirin for preventing stroke in patients with atrial fibrillation.Ann Intern Med 2007;147:590-592.

Healey JS, Hart RG, Pogue J, Pfeffer MA, Hohnloser SH, De Caterina R, Flaker G, Yusuf S, Connolly SJ. Risks and benefits of oral anticoagulation compared with clopidogrel plus aspirin in patients with atrial fibrillation according to stroke risk: the atrial fibrillation clopidogrel trial with irbesartan for prevention of vascular events (ACTIVE-W). Stroke 2008;39:1482-1486.

Hohnloser SH, Pajitnev D, Pogue J, et al. Incidence of stroke in paroxysmal versus sustained atrial fibrillation in patients taking oral anticoagulation or combined antiplatelet therapy. An ACTIVE W substudy. J Am Coll Cardiol 2007;50:2156-2161.

Kamel H, Hegde M, Johnson DR, Gage BF, Johnston SC. Cost-effectiveness of outpatient cardiac monitoring to detect atrial fibrillation after ischemic stroke. Stroke 2010;41:1514-1520.

Kamel H, Johnson DR, Hegde M, Go AS, Sidney S, Sorel M, Hills NK, Johnston SC. Detection of atrial fibrillation after stroke and the risk of recurrent stroke. J Stroke Cerebrovasc Dis 2011 [Epub ahead of print May 3rd]

Kirchhof P, Auricchio A, Bax J, et al. Outcome parameters for trials in atrial fibrillation: executive summary. European Heart J 2007;28:2803.2817.

Liao J, Khalid Z, Scallan C, Morillo C, O'Donnell M. Noninvasive cardiac monitoring for detecting paroxysmal atrial fibrillation or flutter after acute ischemic stroke. A systematic review. Stroke 2007;38:2935-2940.

Ogilvie IM, Newton N, Welner SA, Cowell W, Lip GYH. Underuse of oral anticoagulatns in atrial fibrillation: a systematic review. Am J Med 2010;123:638-645.

Oppenheimer S. Cerebrogenic cardiac arrhythmias: cortical lateralization and clinical significance. Clin Auton Res. 2006 Feb;16(1):6-11.

Schulz KF, Altman DG, Moher D, for the CONSORT Group. Moher D et al. CONSORT 2010 statement: Updated guidelines for reporting parallel group randomized trials. J Pharmacol Pharmacother 2010;1:100–107.

Roten L, Schilling M, Häberlin A, Seiler J, Schwick NG, Fuhrer J, Delacrétaz E, Tanner H. Is 7-day event triggered ECG recording equivalent to 7-day Holter ECG recording for atrial fibrillation screening? Heart. 2012 Apr;98(8):645-9.
Seet RC, Friedman PA, Rabinstein AA. Prolonged Rhythm Monitoring for the Detection of Occult Paroxysmal Atrial Fibrillation in Ischemic Stroke of Unknown Cause. Circulation. 2011:124(4):477-86.
Stahrenberg R, Weber-Krüger M, Seegers J, Edelmann F, Lahno R, Haase B, Mende M, Wohlfahrt J, Kermer P, Vollmann D, Hasenfuß G, Gröschel K, Wachter R. Enhanced detection of paroxysmal atrial fibrillation by early and prolonged continuous Holter monitoring in patients with cerebral ischemia presenting in sinus rhythm. Stroke 2010;41:2884-2888.
Wachter R, Stahrenberg R, Gröschel K. Letter by Wachter et al. Regarding article "Cost-effectiveness of outpatient cardiac monitoring to detect atrial fibrillation to detect ischemic stroke. Stroke 2011;42:e36.

18 APPENDIX
