# Supplementary material for: The cardiac diagnostic work-up in stroke patients—A subanalysis of the Find-AFRANDOMISED trial
Source: PLoS One. 2019 May 9;14(5):e0216530. doi: 10.1371/journal.pone.0216530 (PMC6508702; doi:10.1371/journal.pone.0216530)
Supplement: S1 Table — (DOC) [file pone.0216530.s002.doc]

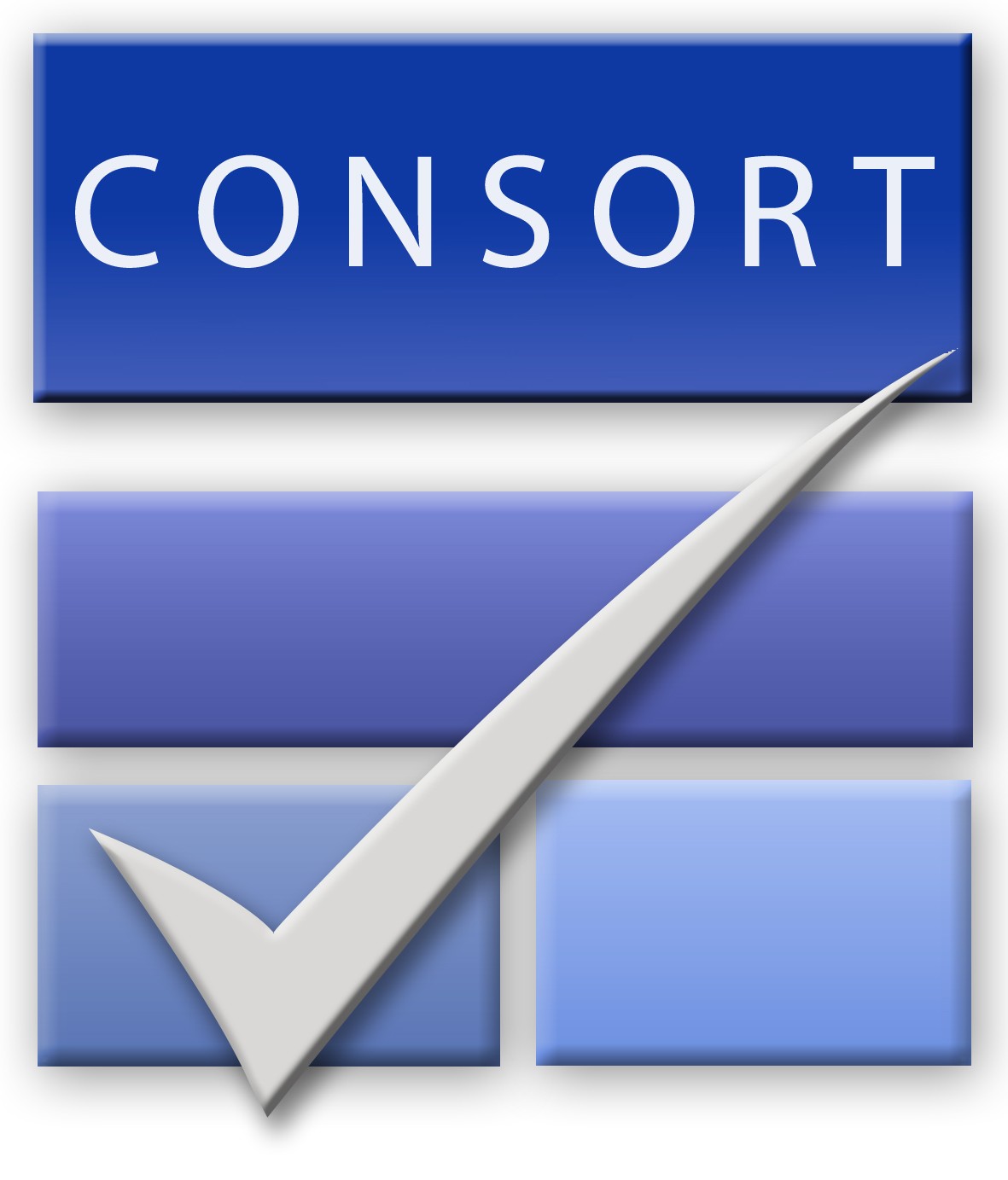
CONSORT 2010 checklist of information to include when reporting a randomised trial*

| Section/Topic | Item No | Checklist item | Reported on page No |
| --- | --- | --- | --- |
| Title and abstract | | | |
|  | 1a | Identification as a randomised trial in the title | 3 |
|  | 1b | Structured summary of trial design, methods, results, and conclusions (for specific guidance see CONSORT for abstracts) | 3 |
| Introduction | | | |
| Background and objectives | 2a | Scientific background and explanation of rationale | 9 |
|  | 2b | Specific objectives or hypotheses | 9 |
| Methods | | | |
| Trial design | 3a | Description of trial design (such as parallel, factorial) including allocation ratio | 10 |
|  | 3b | Important changes to methods after trial commencement (such as eligibility criteria), with reasons | 10 |
| Participants | 4a | Eligibility criteria for participants | 10 |
|  | 4b | Settings and locations where the data were collected | 11 |
| Interventions | 5 | The interventions for each group with sufficient details to allow replication, including how and when they were actually administered | 11 |
| Outcomes | 6a | Completely defined pre-specified primary and secondary outcome measures, including how and when they were assessed | 12 |
|  | 6b | Any changes to trial outcomes after the trial commenced, with reasons | 12 |
| Sample size | 7a | How sample size was determined | 13 |
|  | 7b | When applicable, explanation of any interim analyses and stopping guidelines | 13 |
| Randomisation: |  |  |  |
| Sequence generation | 8a | Method used to generate the random allocation sequence | 13 |
|  | 8b | Type of randomisation; details of any restriction (such as blocking and block size) | 13 |
| Allocation concealment mechanism | 9 | Mechanism used to implement the random allocation sequence (such as sequentially numbered containers), describing any steps taken to conceal the sequence until interventions were assigned | 14 |
| Implementation | 10 | Who generated the random allocation sequence, who enrolled participants, and who assigned participants to interventions | 14 |
| Blinding | 11a | If done, who was blinded after assignment to interventions (for example, participants, care providers, those assessing outcomes) and how | 14 |
|  | 11b | If relevant, description of the similarity of interventions | 14 |
| Statistical methods | 12a | Statistical methods used to compare groups for primary and secondary outcomes | 15 |
|  | 12b | Methods for additional analyses, such as subgroup analyses and adjusted analyses | 15 |
| Results | | | |
| Participant flow (a diagram is strongly recommended) | 13a | For each group, the numbers of participants who were randomly assigned, received intended treatment, and were analysed for the primary outcome | 16 |
|  | 13b | For each group, losses and exclusions after randomisation, together with reasons | 16 |
| Recruitment | 14a | Dates defining the periods of recruitment and follow-up | 16 |
|  | 14b | Why the trial ended or was stopped | 17 |
| Baseline data | 15 | A table showing baseline demographic and clinical characteristics for each group | 18 |
| Numbers analysed | 16 | For each group, number of participants (denominator) included in each analysis and whether the analysis was by original assigned groups | 20 |
| Outcomes and estimation | 17a | For each primary and secondary outcome, results for each group, and the estimated effect size and its precision (such as 95% confidence interval) | 21 |
|  | 17b | For binary outcomes, presentation of both absolute and relative effect sizes is recommended | 21 |
| Ancillary analyses | 18 | Results of any other analyses performed, including subgroup analyses and adjusted analyses, distinguishing pre-specified from exploratory | 22 |
| Harms | 19 | All important harms or unintended effects in each group (for specific guidance see CONSORT for harms) | 23 |
| Discussion | | | |
| Limitations | 20 | Trial limitations, addressing sources of potential bias, imprecision, and, if relevant, multiplicity of analyses | 23 |
| Generalisability | 21 | Generalisability (external validity, applicability) of the trial findings | 24 |
| Interpretation | 22 | Interpretation consistent with results, balancing benefits and harms, and considering other relevant evidence | 24 |
| Other information | | |  |
| Registration | 23 | Registration number and name of trial registry | 24 |
| Protocol | 24 | Where the full trial protocol can be accessed, if available | 25 |
| Funding | 25 | Sources of funding and other support (such as supply of drugs), role of funders | 25 |

*We strongly recommend reading this statement in conjunction with the CONSORT 2010 Explanation and Elaboration for important clarifications on all the items. If relevant, we also recommend reading CONSORT extensions for cluster randomised trials, non-inferiority and equivalence trials, non-pharmacological treatments, herbal interventions, and pragmatic trials. Additional extensions are forthcoming: for those and for up to date references relevant to this checklist, see [www.consort-statement.org](http://www.consort-statement.org/).

Title and Abstract

1a Identification as a randomised trial in the title

Find-AF_randomised_ was a prospective, randomised, controlled study to determine the detection of atrial fibrillation by prolonged and enhanced Holter-ECG monitoring as compared to usual care in stroke patients. The current sub-analysis compared therapeutic consequences based on pathologic findings of both ECG monitoring modalities and of TTE and TEE and investigated the impact of pathologic findings in both diagnostic modalities on the prognosis represented by one-year mortality.

1b Structured summary of trial design, methods, results, and conclusions

| **1. Item** | **2. Description** |
| --- | --- |
| Authors | Corresponding author: Prof. Dr. med. Rolf Wachter, Clinic and Policlinic for Cardiology, University Hospital Leipzig, 04103 Leipzig, Germany, Phone +49-341-97-12650, Fax +49-341-97-12659, E-mail Rolf.Wachter@medizin.uni-leipzig.de |
| Trial design | Find-AF_randomised_:  Allocation: Randomized (ECG monitoring), non-randomized (echocardiography)  Intervention Model: Parallel Assignment (ECG monitoring)  Masking: None (Open Label)  Primary Purpose: Prevention  The current subanalysis investigated the pathologies and the corresponding therapeutic consequences of two randomized diagnostic modalities (enhanced and prolonged Holter-ECG monitoring and usual care (Holter-) ECG monitoring). Furthermore, we evaluated the pathologies and their therapeutic consequences detected by two non-randomized diagnostic modalities (transthoracic and transesophageal echocardiography). In addition we compare the one-year mortality in patients with or without pathologies in either ECG monitoring or echocardiography. |
| Methods: |  |
| Participants | | Ages Eligible for Study: | 60 Years and older   (Adult, Older Adult) | | --- | --- | | Sexes Eligible for Study: | All | | Accepts Healthy Volunteers: | No |   Inclusion Criteria:   - Recent cerebral ischemia defined as stroke (sudden focal neurologic deficit lasting > 24h consistent with the territory of a major cerebral artery and categorised as ischemic) and/or a corresponding lesion on brain imaging. - Stroke symptoms started ≤ 7 days ago. - Age ≥ 60 years. - Modified Rankin scale ≤ 2 (prior to index event).   Exclusion Criteria:   - Known history of atrial fibrillation/flutter or atrial fibrillation/flutter on admission ECG. - Indication for oral anticoagulation at randomisation. - Absolute contra-indication against oral anticoagulation at randomisation. - Intracerebral bleeding in medical history. - Patient scheduled for Holter-ECG or cardiac Event-Recording monitoring ≥ 48 hours. - Significant carotid artery or vertebral artery stenosis > 50% (NASCET classification), significant intracranial artery stenosis suspicious of atherosclerotic origin or acute arterial dissection explanatory of stroke symptoms. - Implanted pacemaker device or cardioverter/defibrillator. - Life expectancy < 1 year for reasons other than stroke (e.g. metastatic cancer). - Concomitant participation in other controlled randomised trial. |
| Interventions | Find-AF_randomised_:  Experimental arm: prolonged ECG monitoring: 10-day Holter ECG at months 0, 3 and 6  Standard care arm: Usual care according to current guidelines (minimum of 24 hours of cardiac monitoring).  The current subanalysis had no interventions. |
| Objective | Find-AF_randomised_:  The purpose of this study is to assess whether repeated enhanced and prolonged ECG monitoring after ischemic stroke results in a higher detection of atrial fibrillation (/flutter) compared to usual care (at least 24 hour of cardiac monitoring).  In this subanalysis of the Find-AF_RANDOMISED_ trial we aimed to analyse the impact of pathologies detected by echocardiography and ECG monitoring on therapeutic decisions and prognosis. |
| Outcome | The primary outcome measure of the current subanalysis was the number of therapeutic decisions based on the four different diagnostic modalities (enhanced and prolonged Holter-ECG monitoring vs. usual care (Holter-) ECG monitoring vs. transthoracic echocardiography vs. transesophageal echocardiography).  Secondary outcome measures were the number and type of pathologies detected by the four diagnostic modalities and the one-year mortality of patients with or without pathologies in ECG monitoring or echocardiography. |
| Randomisation | Find-AF_RANDOMISED_:  Patients were randomized 1:1 to either enhanced and prolonged Holter ECG monitoring (10 days at  baseline and after 3 and 6 months) or standard of care (≥24-hour continuous ECG monitoring, according to current stroke  guidelines).  As you can see above patients of the FIND-AF_RANDOMISED_ were randomised to enhanced and prolonged Holter-ECG monitoring of usual care ECG monitoring. Patients were not randomised to both echocardiographic modalities. The type of echocardiography was chosen by the treating physicians according to local in-house policies. |
| Blinding (masking) | The adjudication committee, that evaluated all episodes potentially resulting in a new diagnosis of AF/flutter after randomization in both trial arms, was blinded to all clinical data. Patients and their treating physicians were not blinded to the randomisation arms. |
| Results: |  |
| Numbers randomised | 402 patients were randomised. Four patients were randomised erroneously (two to each group, three patients with AF prior to randomisation and one patient with severe ipsilateral carotid artery stenosis) and were excluded from the final analysis. 200 patients were assigned to the intervention group and 198 to the control group. |
| Recruitment | Recruitment was stopped on Aug 31, 2014. Aug 31, 2014. The last patient’s final visit was on Sept 16, 2015. |
| Numbers analysed | Enhanced prolonged monitoring: 200 patients   - 7 patients without echocardiography - 113 patients with only TTE - 29 patients with only TEE - 51 patients with TTE and TEE   Usual care: 198 patients   - 8 patients without echocardiography - 115 patients with only TTE - 24 patients with only TEE - 51 patients with TTE and TEE |
| Outcome | ECG:  Pathologies:  Atrial fibrillation: n = 36 (enhanced and prolonged Holter-ECG monitoring: n = 27, usual care (Holter-) ECG monitoring: n = 9; 13.5 % vs. 4.5 %). No potential pacemaker indications were documented.  Therapeutic decisions based on ECG findings:  All 36 patients were treated with oral anticoagulation.  Echocardiography:  Pathologies:   - Hypo- and / or akinesia: n = 44 - PFO and / or ASA: n = 40 - Aortic plaques: n = 34 - LVEF < 50 %: n = 27 - Aortic aneurysm: n = 3 - Severe valve diseases: n = 3 - Valve thrombosis: n = 2 - Left atrial (appendage) or ventricular thrombus: n = 1 - Left ventricular aneurysm: n = 1 - Myxoma: n = 1 - Endocarditis: n = 0   Therapeutic decisions based on echocardiography findings:  - Oral anticoagulation   - PFO and / or ASA: n = 9, based on TEE - Left atrial appendage thrombus: n = 1, based on TEE - ulcerated aortic plaque: n = 1, based on TEE - Valve thrombosis: n = 1, based on TEE   - Operation   - Valve disease (aortic valve stenosis): n = 1, based on TTE - Valve thrombosis: n = 1, based on TEE   - PFO closure: n = 1, based on TEE  Prognosis:  17 study patients died within one year. Of those, 16 received echocardiographic examinations. Three different echocardiographic pathologies were detected in these patients: wall motion abnormalities in six patients, a reduced LVEF and aortic plaques each in three patients. Patients with pathologic echocardiographic findings had a trend towards higher one-year mortality, whereas pathologic ECG findings were not associated with higher one-year mortality (p = 0.093 vs. p = 0.6). |
| Harms | Recurrent strokes after 12 months occurred in five patients in the intervention group versus nine patients in the control  group; and the number of total deaths after 12 months was six in the intervention group versus nine in the control group.  17 study patients died within one year. Three different echocardiographic pathologies were detected in these patients: wall motion abnormalities in six patients, a reduced LVEF and aortic plaques each in three patients. The death of all three patients with the combination of reduced LVEF and wall motion abnormalities was classified as “cardiovascular”. AF was diagnosed in three of the patients who died within one year after randomization, two by EPM, one by routine Holter-ECG. Of those, two patients additionally suffered from the combination of reduced LVEF and wall motion abnormalities. |
| Conclusions | Enhanced and prolonged monitoring initiated early in patients with acute ischaemic stroke aged 60 years or older was the diagnostic tool, which was most effective in our cohort, followed by TEE, usual care (Holter-) ECG monitoring and TTE. Although echocardiography rarely result in therapeutic changes, it can identify a patient group at high cardiovascular risk. |
| Trial registration | http://www.clinicaltrials.gov. Unique identifier: NC NCT01855035. |
| Funding | IFS Göttingen (Institut für anwendungsorientierte Forschung und klinische Studien) acts as the sponsor. The trial is supported by an unrestricted grant from Boehringer Ingelheim. The funder had no role in the trial design and conduct, data analysis or interpretation, or publication and authorship decisions of the report. |

Introduction

Background and objectives

2a Scientific background and explanation of rationale

The standard of care cardiac diagnostic work-up of stroke patients includes continuous monitoring of vital parameters and neurologic status, systematic laboratory testing, cerebral imaging, extra- and transcranial Doppler and duplex sonography, 12-lead ECG and a minimum of 24-hour ECG-monitoring. In the past few years evidence accumulated that enhanced and prolonged Holter-ECG monitoring can reveal a much higher rate of (paroxysmal) atrial fibrillation, which usually shifts the secondary stroke prevention to oral anticoagulation, which reduces the recurrent stroke rate significantly. Echocardiography has also the potential of detecting pathologies of crucial therapeutic impact. Apart from cardiac sources of embolism left atrial (appendage) or ventricular thrombus, patent foramen ovale, atrial septum aneurysm, endocarditis or cardiac tumor, echocardiography can also detect cardiac pathologies of relevance for well-being and survival of the patients like wall motion abnormalities or a reduced left ventricular function, which also potentially change therapeutic decisions.

Despite the fact that echocardiography can provide useful information in stroke patients the indication and optimal echocardiographic approach in the cardiac workup of ischemic stroke are still unclear and not specifically addressed in current AHA/ASA guidelines. Furthermore, therapeutic decisions based on echocardiographic findings in stroke patients often seem to underlie local in-house policies or are reduced to the treatment of pathologies that are directly linked to stroke like cardiac sources of embolism, instead of treating all pathologies that affect the well-being and survival or simply the prognosis of stroke patients.

2b Specific objectives or hypotheses

In this subanalysis of the Find-AF_RANDOMISED_ trial we therefore wanted to investigate in a first step how many and which pathologies were detected by the four diagnostic modalities (enhanced and prolonged Holter-ECG monitoring, usual care (Holter-) ECG monitoring, transthoracic or transesophageal echocardiography) and how often they finally lead to a new therapeutic decision in stroke patients. In a second step we compared the prognosis (one-year mortality) in patients with or without pathologies in either ECG monitoring (enhanced and prolonged and usual care ECG monitoring) or echocardiography (transthoracic and transesophageal echocardiography).

Methods

Trial Design

3a Description of trial design (such as parallel, factorial) including allocation ratio

Find-AF_RANDOMISED_ was an investigator-initiated randomised, controlled, open-label multicentre trial done in four centres in Germany (University Medicine Göttingen [Göttingen], University Medicine Mainz [Mainz], Horst-Schmidt-Kliniken Wiesbaden [Wiesbaden], and Nordwest-Krankenhaus Sanderbusch [Sande]). We assessed atrial fibrillation or atrial flutter and recurrent stroke or transient ischaemic attack (TIA) through independent blinded expert adjudication committees (prospective randomised open-label blinded endpoint evaluation [PROBE] design).

In this subanalysis we furthermore evaluated pathologies of potential therapeutic impact detected by either transthoracic or transesophageal echocardiography. Patients were not randomised to one type of echocardiography. The decision which type of echocardiography was performed in a patient was left to the treating physicians according to local in-house policies.

3b Important changes to methods after trial commencement (such as eligibility criteria), with reasons

None.

Participants

4a Eligibility criteria for participants

Inclusion Criteria:

• Recent cerebral ischemia defined as stroke (sudden focal neurologic deficit lasting > 24h consistent with the territory of a major cerebral artery and categorised as ischemic) and/or a corresponding lesion on brain imaging.

• Stroke symptoms started ≤ 7 days ago.

• Age ≥ 60 years.

• Modified Rankin scale ≤ 2 (prior to index event).

Exclusion Criteria:

• Known history of atrial fibrillation/flutter or atrial fibrillation/flutter on admission ECG.

• Indication for oral anticoagulation at randomisation.

• Absolute contra-indication against oral anticoagulation at randomisation.

• Intracerebral bleeding in medical history.

• Patient scheduled for Holter-ECG or cardiac Event-Recording monitoring ≥ 48 hours.

• Significant carotid artery or vertebral artery stenosis > 50% (NASCET classification), significant intracranial artery stenosis suspicious of atherosclerotic origin or acute arterial dissection explanatory of stroke symptoms.

• Implanted pacemaker device or cardioverter/defibrillator.

• Life expectancy < 1 year for reasons other than stroke (e.g. metastatic cancer).

• Concomitant participation in other controlled randomised trial.

4b Settings and locations where the data were collected

All documented data were introduced into a secure electronic clinical report form (eCRF) database, which was provided, managed, and secured by IFS (Institut fuer anwendungsorientierte Forschung und klinische Studien GmbH) Göttingen.

Interventions

5 Find-AF_RANDOMISED_ compares two diagnostic strategies: “Repeated enhanced and prolonged Holter ECG monitoring” (3 × 10 days, first recording early after index event, second after 3 months, and third after 6 month) versus “standard-of-care” procedures, which, according to current stroke guidelines, include a minimum of 24 hours of continuous ECG monitoring (stroke unit monitoring and/or Holter ECG, according to local standards). Since this is a subanalysis of Find-AF_RANDOMISED_ there were no further interventions.


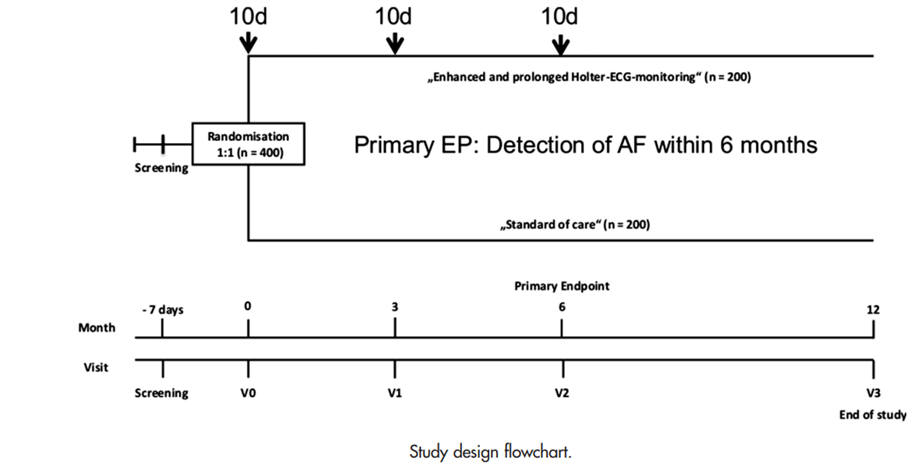


Outcomes

6a Primary Outcome Measures:

The primary outcome measure of the current subanalysis was the number of therapeutic decisions based on the four different diagnostic modalities (enhanced and prolonged Holter-ECG monitoring vs. usual care (Holter-) ECG monitoring vs. transthoracic echocardiography vs. transesophageal echocardiography).

Secondary outcome measures were the number and type of pathologies detected by the four diagnostic modalities and the one-year mortality of patients with or without pathologies in ECG monitoring or echocardiography.

6b Any changes to trial outcomes after the trial commenced, with reasons

None.

Sample Size

7a How sample size was determined

The sample size discussion of FIND-AF_RANDOMISED_ is based on the forerunner trial “Find-AF.” Although the evaluation of every 24-hour Holter ECG interval yielded 3% to 5% newly diagnosed paroxysmal AF, the 7-day Holter ECG detected 12.5%. With an additional 23 days of Holter recordings, we estimate the detection rate in the intervention arm to be at least 15%. We assume a dropout rate of 15%; thus, 340 of the 400 patients will be analysed. This sample size would provide 83% power to distinguish between 15% (within the intervention arm) versus 5% (within the control arm) paroxysmal AF and 98% power to distinguish between detection rates of 20% versus 5%. Since this a subanalysis of FIND-AF_RANDOMISED_ there was no more detailed sample size calculation.

7b When applicable, explanation of any interim analyses and stopping guidelines

Not applicable.

Randomisation

Sequence generation

8a Method used to generate the random allocation sequence

Patients were randomised in a 1:1 ratio to either “repeated enhanced and prolonged ECG monitoring” or “standard-of-care.” Patients were not randomised to echocardiography types. Neither the comparison of prognosis between the groups of patients with or without pathologies in either ECG monitoring nor echocardiography was randomised.

8b Type of randomisation; details of any restriction (such as blocking and block size)

Permuted-block randomisation of ECG types with block sizes of 2, 4, 6, and 8 was stratified by each participating study centre.

Allocation concealment mechanism

9 Mechanism used to implement the random allocation sequence (such as sequentially numbered containers), describing any steps taken to conceal the sequence until interventions were assigned

Randomisation was done using sequentially numbered, opaque sealed envelopes, to be opened in consecutive order.

Implementation

10 Who generated the random allocation sequence, who enrolled participants, and who assigned participants to interventions

The computer-generated random allocation sequence was provided by the Institut für anwendungsorientierte Forschung und klinische Studien (IFS) Göttingen, Germany. Patients were enrolled by study personell.

Blinding

11a If done, who was blinded after assignment to interventions (for example, participants, care providers, those assessing outcomes) and how

All episodes potentially resulting in a new diagnosis of AF/flutter after randomization, that is, those detected by means of study-specific Holter ECG or event monitoring, as well as those recorded during routine diagnostic workup (including ECGs from external centers) in both trials arms, will be evaluated by an independent AF end point adjudication committee, blinded to all clinical data. Participants and the treating physicians were not blinded to enhanced and prolonged Holter-ECG monitoring or usual care (Holter-) ECG monitoring or transthoracic or transesophageal echocardiography.

11b If relevant, description of the similarity of interventions

Not relevant.

Statistical Methods

12a Statistical methods used to compare groups for primary and secondary outcomes

The primary hypothesis is that enhanced and prolonged monitoring is the most effective diagnostic tool in the cardiac work-up of stroke patients ≥ 60 years. We therefore compared the number of pathologies detected by four diagnostic modalities: (I) enhanced and prolonged Holter-ECG monitoring, (II) usual care ≥ 24 h (Holter-) ECG monitoring, (III) transthoracic echocardiography, and (IV) transesophageal echocardiography and which and how many therapeutic decisions were made based on these findings. Comparisons of means were realized by t-test for independent samples. Median values with the corresponding interquartile range (IQR) were computed for non-normally distributed variables. For a straightforward interpretation we defined the measure "Number Needed to Change Decision" (NNCD) as the average number of patients to be diagnosed in order to encounter one for whom the treatment decision changes. NNCD is the inverse of the absolute frequency. Nonparametric 95% confidence intervals were calculated and tests were performed by simulation (n = 10,000).

All statistical analyses were performed using SPSS version 23.0 and higher (SPSS, Inc.) and R. Significance level for two-tailed tests is defined 0.05.

12b Methods for additional analyses, such as subgroup analyses and adjusted analyses

In a second step we compared the one-year mortality of patients with or without pathologies in either ECG monitoring or echocardiography to evaluate the impact of the pathologies detected. One-year survival data for patients with or without pathologic echocardiographic or ECG findings were depicted using the Kaplan-Meier method. To adjust for age and centre heterogeneity, we fitted mixed linear Cox model for time-to-event data with random intercept for centre.

Results

Participant flow

13 a For each group, the numbers of participants who were randomly assigned, received intended treatment, and were analysed for the primary outcome

200 patients randomised to enhanced and prolonged Holter-ECG monitoring, of those

- 7 patients without echocardiography
- 113 patients with only TTE
- 29 patients with only TEE
- 51 patients with TTE and TEE

198 patients randomised to usual care (Holter-) ECG monitoring

- 8 patients without echocardiography
- 115 patients with only TTE
- 24 patients with only TEE
- 51 patients with TTE and TEE

13 b For each group, losses and exclusions after randomisation, together with reasons

Screening of 2848 patients with the diagnosis I63.x

Randomisation of 402 patients

Erroneous randomisation of 4 patients (3 x AF, 1 x ICA stenosis, 2 patients were part of the enhanced and prolonged Holter-ECG monitoring group and 2 patients belonged to the usual care (Holter-) ECG monitoring group)

Recruitment

14 a Dates defining the periods of recruitment and follow-up

Patients were included within 7 days after the index stroke. Clinical follow-up visits took place 3, 6, and 12 months after the index event. Information on recent morbidity (including a new diagnosis of AF/flutter), current medication, adverse events, and guideline adherence is collected. Since echocardiography was done according to local in-house policies there was no standardized procedure concerning the type of echocardiograph or its time-point.


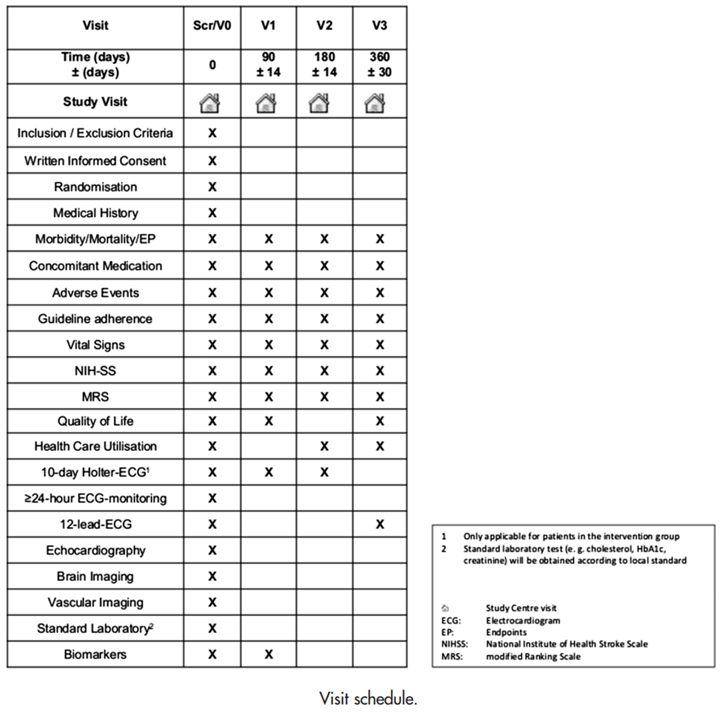


14 b Why the trial ended or was stopped

The trial ended as planned after all patients had completed 12 months of follow-up, and the last patient’s final visit was on Sept 16, 2015.

Basline data

15 A table showing baseline demographic and clinical characteristics for each group


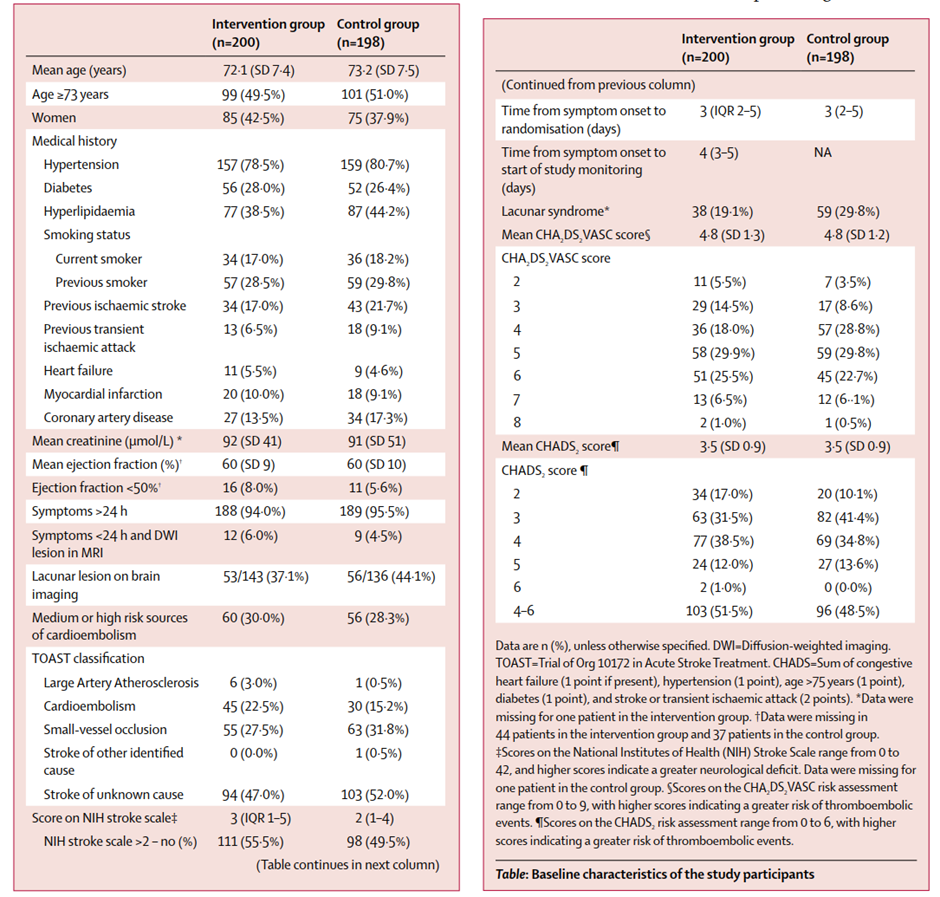


|  | | | All  n = 398 | EPM  n = 200 | | | | Usual care  n = 198 | | | | |
| --- | --- | --- | --- | --- | --- | --- | --- | --- | --- | --- | --- | --- |
|  | | |  | no echocardio-graphy n = 7 | only TTE done  n = 113 | only TEE done  n = 29 | TTE and TEE done n = 51 | | no echocardio-graphy n = 8 | only TTE done n = 115 | only TEE done  n = 24 | TTE and TEE done n = 51 |
| Mean age (years) | | | 72.7 (SD 7.5) | 74.7 (SD 5.5) | 74.5 (SD 7.1) | 68.7 (SD 6.6) | 68.5 (SD 6.6) | | 76.1 (SD 7.8) | 74.8 (SD 7.4) | 72.4 (SD 8.1) | 69.7 (SD 6.4) |
| Female sex | | | 160 (40.1%) | 4 (57.1%) | 51 (45.1%) | 10 (34.5%) | 20 (39.2%) | | 5 (62.5%) | 45 (39.1 %) | 5 (20.8%) | 20 (39.2%) |
| Medical history | | |  |  |  |  |  | |  |  |  |  |
|  | Art. hypertension | | 316 (79.2%) | 6 (85.7%) | 95 (84.1%) | 21 (72.4%) | 35 (68.6%) | | 8 (100.0%) | 92 (80.0%) | 17 (70.8%) | 42 (82.4%) |
|  | Diabetes mellitus | | 108 (27.1%) | 4 (57.1%) | 37 (32.7%) | 6 (20.7%) | 9 (17.6%) | | 2 (25.0%) | 28 (24.3%) | 7 (29.2%) | 15 (29.4%) |
|  | Hyperlipidemia | | 164 (41.1%) | 3 (42.9%) | 49 (43.4%) | 5 (17.2%) | 20 (39.2%) | | 4 (50.0%) | 48 (41.7%) | 10 (41.7%) | 25 (49.0%) |
|  | Smoking status | |  |  |  |  |  | |  |  |  |  |
|  |  | Current smoker | 70 (17.5%) | 1 (14.3%) | 15 (13.3%) | 7 (24.1%) | 11 (21.6%) | | 1 (12.5%) | 16 (13.9%) | 6 (25.0%) | 13 (25.5%) |
|  |  | Previous smoker | 116 (29.1%) | 2 (28.6%) | 27 (23.9%) | 10 (34.5%) | 18 (35.5%) | | 2 (25.0%) | 36 (31.3%) | 9 (37.5%) | 12 (23.5%) |
|  | Previous ischemic stroke | | 77 (19.3%) | 1 (14.3%) | 20 (17.7%) | 5 (17.2%) | 8 (15.7%) | | 3 (37.5%) | 24 (20.9%) | 2 (8.3%) | 14 (27.5%) |
|  | Previous TIA | | 31 (7.8%) | 0 (0.0%) | 8 (7.1%) | 1 (3.4%) | 4 (7.8%) | | 1 (12.5%) | 12 (10.4%) | 1 (4.2%) | 4 (7.8%) |
|  | Myocardial infarction | | 38 (9.5%) | 1 (14.3%) | 14 (12.4%) | 3 (10.3%) | 2 (3.9%) | | 1 (12.5%) | 11 (9.6%) | 1 (4.2%) | 5 (9.8%) |
|  | Coronary artery disease | | 61 (15.3%) | 1 (14.3%) | 16 (14.2%) | 6 (20.7%) | 4 (7.8%) | | 2 (25.0%) | 15 (13.0%) | 6 (25.0%) | 11 (21.6%) |
| TOAST classification | | |  |  |  |  |  | |  |  |  |  |
|  | Large artery sclerosis | | 7 (1.8%) | 0 (0.0%) | 4 (3.5%) | 0 (0.0%) | 2 (3.9%) | | 0 (0.0%) | 1 (0.9%) | 0 (0.0%) | 0 (0.0%) |
|  | Cardioembolism | | 75 (18.8%) | 0 (0.0%) | 19 (14.8%) | 10 (34.5%) | 16 (31.4%) | | 0 (0.0%) | 13 (11.3%) | 5 (20.8%) | 12 (23.5%) |
|  | Small vessel occlusion | | 118 (29.6%) | 0 (0.0%) | 35 (31.0%) | 8 (27.6%) | 12 (23.5%) | | 0 (0.0%) | 40 (34.8%) | 0 (0.0%) | 17 (33.3%) |
|  | Stroke or other identified cause | | 1 (0.3%) | 0 (0.0%) | 0 (0.0%) | 0 (0.0%) | 0 (0.0%) | | 0 (0.0%) | 1 (0.9%) | 0 (0.0%) | 0 (0.0%) |
|  | Stroke of unknown cause | | 197 (49.4%) | 7 (100.0%) | 55 (48.7%) | 11 (37.9%) | 21 (41.2%) | | 8 (100.0%) | 60 (52.2%) | 13 (54.2%) | 22 (43.1%) |
| Score on NIHSS | | |  |  |  |  |  | |  |  |  |  |
|  | Median NIHSS (IQR) | | 3 (IQR 1-5) | 2 (IQR 1-6) | 3 (IQR 1-6) | 3 (IQR 2-5) | 3 (IQR 1-5) | | 3 (IQR 1-8) | 2 (IQR 1-4) | 2 (IQR 1-3) | 3 (IQR 1-5) |

Numbers analysed

16 For each group, number of participants (denominator) included in each analysis and whether the analysis was by original assigned groups

FindAF_randomised_:

Enhanced prolonged monitoring group: 202 patients were included in the intention-to-monitor analysis

Usual care group: 200 patients were included in the intention-to-monitor analysis

Present subanalysis:

Enhanced prolonged monitoring group: n = 200, no echocardiography: n = 7; only TTE: n =113, only TEE: n = 29, TTE and TEE: n = 51

Usual care group: n = 198, no echocardiography: n = 8; only TTE: n =115, only TEE: n = 24, TTE and TEE: n = 51

Outcomes and estimation

17a/b For each primary and secondary outcome, results for each group, and the estimated effect size and its precision (such as 95% confidence interval). For binary outcomes, presentation of both absolute and relative effect sizes is recommended

ECG monitoring revealed 36 pathologies

- AF n = 36,

enhanced and prolonged Holter-ECG monitoring: n = 27

usual care (Holter-) ECG monitoring n = 9

- No potential pace maker indications were found.

Therapeutic decisions based on ECG findings:

All 36 AF patients were treated with oral anticoagulation.

Echocardiography revealed 179 pathologies in 112 patients (89 detected by TEE and 90 detected by TTE). In detail, we found:

- Hypo- and / or akinesia: n = 44
- PFO and / or ASA: n = 40
- Aortic plaques: n = 34
- LVEF < 50 %: n = 27
- Aortic aneurysm: n = 3
- Severe valve diseases: n = 3
- Valve thrombosis: n = 2
- Left atrial (appendage) or ventricular thrombus: n = 1
- Left ventricular aneurysm: n = 1
- Myxoma: n = 1
- Endocarditis: n = 0

Therapeutic decisions based on echocardiography findings:

- Oral anticoagulation
- PFO and / or ASA: n = 9, based on TEE
- Left atrial appendage thrombus: n = 1, based on TEE
- ulcerated aortic plaque: n = 1, based on TEE
- Valve thrombosis: n = 1, based on TEE
- Operation
- Valve disease (aortic valve stenosis): n = 1, based on TTE
- Valve thrombosis: n = 1, based on TEE

- PFO closure: n = 1, based on TEE

The one year mortality was numerical, but not statistically significant higher in patients with pathologies in ECG monitoring or echocardiography. The age-adjusted p-value was 0.076 in patients with or without echocardiographic pathologies and 0.71 in patients with or without ECG monitoring.

Ancillary analyses

18 Results of any other analyses performed, including subgroup analyses and adjusted analyses, distinguishing pre-specified from exploratory

50.3% received EPM and 49.7% routine ECG monitoring. 82.9 % underwent transthoracic echocardiography (TTE), 38.9 % transesophageal echocardiography (TEE) and 25.6 % both procedures. 14/89 TEE pathologies and 1/90 TTE pathology led to a change in therapy, resulting in a number needed to change decision (NNCD) of 12 and 334, respectively. In comparison, EPM found atrial fibrillation (AF) in 27 of 200 patients, and routine ECG monitoring in twelve of 198 patients, leading to therapeutic changes in all patients (NNCD 8 and 17, respectively). We found a trend towards a higher age-adjusted one-year mortality in patients with pathologic echocardiographic findings (p = 0.076).

Harms

19 All important harms or unintended effects in each group (for specific guidance see CONSORT for harms)

Within 12 months of follow-up, eight (five recurrent strokes and three TIAs) patients in the intervention group and 14 (nine recurrent strokes and five TIAs) patients in the control group had recurrent cerebral ischaemic events. Two patients (one in each group) had two recurrent strokes per person. Kaplan-Meier stroke rate was 5.4% in the control group and 3.7% in the intervention group, difference 1.7% (95% CI –2.5 to 5.9, p=0.46). No cases of systemic embolism occurred in either group. 17 study patients died within one year. Of those, 16 received echocardiographic examinations. Three different echocardiographic pathologies were detected in these patients: wall motion abnormalities in six patients, a reduced LVEF and aortic plaques each in three patients. Patients with pathologic echocardiographic findings had a trend towards higher one-year mortality, whereas pathologic ECG findings were not associated with a higher age-adjusted one-year mortality (p = 0.076 vs. p = 0.71, see Fig 4). The death of all three patients with the combination of reduced LVEF and wall motion abnormalities was classified as “cardiovascular”. AF was diagnosed in three of the patients who died within one year after randomization, two by EPM, one by routine Holter-ECG. Of those, two patients additionally suffered from the combination of reduced LVEF and wall motion abnormalities.

Discussion

Limitations

20 Trial limitations, addressing sources of potential bias, imprecision, and, if relevant, multiplicity of analyses

The FIND-AF_RANDOMISED_ trial was done in Germany and it cannot be ruled out that results would have differed in other countries, health systems, or in people of different ethnic origins. The participation rate during the second and third Holter-ECG was lower (116 of 170 patients after 3 months and 100 of 153 patients after 6 months, respectively) than during the first Holter-ECG and the yield of repeated monitoring could be higher, assuming better compliance. This problem might be solved by the use of more comfortable devices, such as adhesive ECG-patches. More prolonged monitoring approaches, such as implanted devices, might have yielded an even higher rate of atrial fibrillation in this cohort. The classification of baseline strokes into the Trial of Org 10172 in Acute Stroke Treatment (TOAST) criteria was based on information provided by the local investigators and a structured analysis from an independent panel might have led to different results.

In this present subanalysis, echocardiography rates differed widely between the centres which limits generalisability and may reflect selection bias. A study design with a standardized algorithm and a randomization of the two echocardiographic modalities would have been advantageous for the present question. Additionally, although Find-AF_RANDOMISED_ was a prospective randomised trial, this analysis was not pre-specified and should therefore be considered descriptive and hypothesis-generating.

Generalisability

21 Generalisability (external validity, applicability) of the trial findings

Our results were obtained in patients aged ≥ 60 years. The prevalence of PFO is likely to be higher in younger stroke patients, whereas the prevalence of AF is supposed to be higher in older patients, which might partly explain the diagnostic superiority of repeated and prolonged Holter-ECG monitoring in our study cohort.

Interpretation

22 Interpretation consistent with results, balancing benefits and harms, and considering other relevant evidence

This subanalysis of the Find-AF_RANDOMISED_ trial showed that enhanced and prolonged ECG-monitoring is the key tool in the cardiac workup of ischemic stroke in patients older than 60 years and should therefore play a more prominent role in future guidelines. Prolonged and enhanced monitoring should be used in all patients with stroke if the detection of atrial fibrillation would lead to anticoagulation therapy. Echocardiography of any type helps to identify a subgroup of stroke patients at high cardiovascular risk. A better neurocardiologic diagnostic and therapeutic collaboration and further appropriate trials are warranted.

Other Information

Registration

23 Registration number and name of trial registry

Unique identifier of Find-AF_RANDOMISED_: NC NCT01855035. http://www.clinicaltrials.gov.

Protocol

24 Where the full trial protocol can be accessed, if available

<https://clinicaltrials.gov/ct2/show/NCT01855035> (Find-AF_RANDOMISED_)

Funding

25 Sources of funding and other support (such as supply of drugs), role of funders

IFS Göttingen (Institut für anwendungsorientierte Forschung und klinische Studien) acts as the sponsor. The trial is supported by an unrestricted grant from Boehringer Ingelheim. The funder had no role in the trial design and conduct, data analysis or interpretation, or publication and authorship decisions of the report.
